# The Evolution of Psychological and Behavioral Consequences of Self-Isolation During Lockdown: A Longitudinal Study Across United Kingdom and Italy

**DOI:** 10.3389/fpsyt.2022.826277

**Published:** 2022-06-01

**Authors:** Francesca Zaninotto, Francesco Bossi, Philip Terry, Massimo Riccaboni, Giulia Galli

**Affiliations:** ^1^Department of Psychology, Kingston University, Kingston upon Thames, United Kingdom; ^2^MoMiLab, IMT School for Advanced Studies Lucca, Lucca, Italy; ^3^Axes, IMT School for Advanced Studies Lucca, Lucca, Italy

**Keywords:** COVID-19, lockdown, pandemic, longitudinal, stress, wellbeing, anxiety, depression

## Abstract

**Introduction:**

Several countries imposed nationwide or partial lockdowns to limit the spread of COVID-19 and avoid overwhelming hospitals and intensive care units. Lockdown may involve restriction of movement, stay-at-home orders and self-isolation, which may have dramatic consequences on mental health. Recent studies demonstrated that the negative impact of lockdown restrictions depends on a wide range of psychological and socio-demographic factors.

**Aims:**

This longitudinal study aimed to understand how internal factors such as personality and mindfulness traits, and external factors, such as daily habits and house features, affect anxiety, depression and general wellbeing indicators, as well as cognitive functions, during the course of a lockdown.

**Methods:**

To address these questions, 96 participants in Italy and the United Kingdom filled out a survey, once a week for 4 weeks, during the first-wave lockdowns. The survey included questions related to their habits and features of the house, as well as validated questionnaires to measure personality traits, mindful attitude and post-traumatic symptoms. Indicators of wellbeing were the affective state, anxiety, stress and psychopathological indices. We also measured the emotional impact of the pandemic on cognitive ability by using two online behavioral tasks [emotional Stroop task (EST) and visual search].

**Results:**

We found that internal factors influenced participants’ wellbeing during the first week of the study, while external factors affected participants in the last weeks. In the first week, internal variables such as openness, conscientiousness and being non-judgmental toward one’s own thoughts and emotions were positively associated with wellbeing; instead, neuroticism and the tendency to observe and describe one’s own thoughts and emotions had detrimental effects on wellbeing. Toward the end of the study, external variables such as watching television and movies, browsing the internet, walking the dog, and having a balcony showed a protective value, while social networking and engaging in video calls predicted lower values of wellbeing. We did not find any effects of wellbeing on cognitive functioning.

**Conclusion:**

Recognizing specific traits and habits affecting individuals’ wellbeing (in both short and long terms) during social isolation is crucial to identify people at risk of developing psychological distress and help refine current guidelines to alleviate the psychological consequences of prolonged lockdowns.

## Introduction

Coronavirus (COVID-19) was first declared a public health emergency of international concern in January 2020 and then confirmed as a global pandemic in March 2020 (1). On the 13th March 2020, Italy imposed a total lockdown to manage the spread of Coronavirus and to prevent hospitals and intensive care units from being overwhelmed. The United Kingdom entered into lockdown on the 23rd March 2020, with a less restrictive strategy compared with the Italian lockdown. People in the United Kingdom were allowed to leave their homes to shop for basic necessities, for medical needs, to exercise once a day (run, walk, or cycle) alone or with members of their households and to travel to and from work. In contrast, the Italian government imposed full confinement, requiring the population to stay at home (108, 109). Physical activity (PA) outside was allowed only if conducted individually and not more than 250 meters from home; non-essential shops were closed and a restrictive derogative travel certificate was mandatory for any travel outside of the home (2).

Even when temporary, lockdown restrictions and self-isolation can have dramatic consequences on people’s health and wellbeing (3); in particular, recent international studies have reported increased symptoms of anxiety, depression, sleep disorders, and psychological distress during lockdown (4–10). The factors that negatively influence the individual include separation from loved ones, loss of freedom, uncertainty over disease status, inadequate supplies, inadequate information, frustration, and boredom. Individuals cope with traumatic experiences in different ways, and in periods of uncertainty and environmental change, the role of personality differences becomes more evident (11, 12). Because there is substantial evidence linking personality traits with depressive symptoms and distress (13), some recent studies have explored the role of personality traits during the first lockdown stage, suggesting that vulnerable factors (neuroticism) and protective factors (extraversion and conscientiousness) can predict mental health status [e.g., (14, 15)].

Very little is known about the factors that may protect mental health during traumatic environmental change. The present longitudinal study, conducted over 4 weeks during the initial stages of lockdown in Italy and the United Kingdom in 2020, aimed to identify resilience factors that might mitigate the negative consequences of lockdown and self-isolation, as well as the risk factors that may worsen mental health outcomes. Participants were tested once a week and they were required to answer a questionnaire exploring indexes of depression, stress, and anxiety, as well as daily routines and habits. The questionnaire was paired with two cognitive tasks investigating emotional interference and visual selective attention. Factors related to personality and mindfulness traits were defined as “internal factors,” while factors indicating daily routine, habits and living conditions were defined as “external factors.”

### Internal Factors: Personality and Mindfulness Traits

#### Personality Traits

One internal factor that can be measured to predict health outcomes in stressful situations is personality. The “Big Five” personality test is based on a five-factor model of personality [extraversion, agreeableness, conscientiousness, neuroticism, and openness; (16)], and it has recently been used to explore the relationship between personality traits and mental health during the COVID-19 outbreak. Neuroticism has been associated mostly with worry and stress (17–19). Also, individuals who scored higher on neuroticism have shown a reduced likelihood of engaging in potential safety behaviors [e.g., searching for COVID-19 symptoms on the internet; (18)]. Lower extraversion and higher neuroticism were also associated with higher stress (20). Cases reported by Nikčević and Spada (15) support the hypothesis that certain Big Five personality traits might be related to generalized anxiety and depressive symptoms during the first stage of lockdown. In their study of 502 participants from the United States, three personality traits (extraversion, agreeableness, and conscientiousness) were negatively associated with generalized anxiety and depressive symptoms. In contrast, neuroticism was directly associated with, and considered as a vulnerability factor for generalized anxiety and depressive symptoms. The study by Nikčević and Spada. (15) supported the hypothesis that agreeableness and extraversion might contribute to activating coping strategies (e.g., connecting with others) during the lockdown, and consequently might be considered protective factors that could mitigate negative affect (15).

#### Mindfulness Traits

A second internal factor that may contribute to predicting health outcomes is mindfulness traits. Mindfulness is defined as the ability to be intentionally aware of the present moment and the capacity to acknowledge both internal experiences and external information using a non-reactive (i.e., letting one’s thoughts and feelings go without focusing or elaborating them) and non-judgmental perspective, i.e., taking a non-judgmental stance toward one’s inner experience (21).

Such traits have been shown to play a significant role in depression vulnerability and emotion self-regulation (22). Previous studies conducted during the first stage of lockdown reported that high levels of mindfulness are associated with lower levels of distress (23) and that mindfulness awareness is associated with more preventative health behaviors (24). Lower mindfulness traits have been associated with increased depression, stress, and anxiety (25). Higher scores for non-judgmental traits predicted lower levels of depression, anxiety and stress, and higher scores for mindfulness awareness predicted lower depressive symptoms (26). A web-based survey of a sample of about 6,000 Italians showed that, during the lockdown, increased levels of mindfulness correlated with decreased levels of distress, and were negatively correlated with the Symptom Checklist [SCL-90; (27)] subscales (measuring psychopathological symptoms); in particular, participants who scored lower on mindfulness traits reported higher obsessive-compulsive symptoms (23).

### External Factors: Daily Habits

Lifestyle and daily habits are external factors that can affect how an individual overcomes stressful situations. Research measuring the daily routines of 670 Italians during the first stages of lockdown showed that a person’s daily routine might have a critical effect on their mental health. In a study by Di Corrado et al. (28), most of the participants continued exercising during the lockdown restrictions, but participants whose habits were disrupted during the lockdown reported higher levels of nervousness. In contrast, participants who maintained their training habits reported feeling more energy, less fatigue, and more calm. In addition, participants who started exercising during the lockdown reported higher levels of happiness. Di Corrado et al. (28) suggested that maintaining regular habits during lockdown might prevent individuals from experiencing psychological and physical distress. However, more recently, contradictory evidence has emerged concerning the role played by PA during the lockdown. Some findings indicate a negative correlation between PA and mental health (29, 30), whereas other studies have reported benefits from keeping a regular PA habit during lockdown (31, 32). A longitudinal study between March and April 2020 in German and French populations showed that PA and exposure to nature were significant predictors of psychological health (33).

Walking is associated with physical wellbeing. Owning and walking a dog is recognized as contributing to human health (34, 35). Dog walking was one of the few activities allowed during the lockdown in Italy and the United Kingdom, consequently motivating people to acquire new puppies (“pandemic puppies”) or to adopt rescue dogs (36). Because walking a dog and being a dog owner have been associated with lower depression, these were categorized as protective factors (37–39).

During the lockdown, people took up or increased their involvement in diverse activities, such as creative hobbies (e.g., cooking, reading, etc.), but also spent more time using digital social platforms to connect with others. Digital activity can be a strategy to cope with stressful events (40). However, there are contradictory findings regarding the effects of social media on individuals’ wellbeing during the lockdown. Recent studies conducted showed that the use of social media during the pandemic was associated with feeling overwhelmed due to information overload related to COVID-19, and had a significant impact on users’ wellbeing (41). In contrast, other studies suggested that social media had a positive effect on individuals’ mental health during lockdown (42, 43). The digital activities considered in this study were: using social media, browsing the web, watching movies and television series, watching the news, and listening to music, radio, or podcasts.

### Evolution of Internal and External Factors Over Time

Most recent attention has focused on the weekly progression of psychological wellbeing over the lockdown. For example, a study investigating the correlation between depressive symptoms and PA in Spain reported that the intensity of PA could predict the intensity and the presence of depressive symptoms (44). Ripoll et al. (45) conducted a longitudinal study over 8 weeks during the lockdown in Spain and reported that psychological wellbeing, life satisfaction and self-perceived health did not remain stable during the duration of the study. Anxiety and depressive symptoms improved after weeks four and five, suggesting that people might show resilience to the negative consequences of lockdown (45). A study conducted at three separate times during the first month of lockdown in Italy suggested an increase in stress, anxiety and life satisfaction levels between the second and the third testing sessions, but a stable depression level over time (46). Another longitudinal study in Italy, comparing participants’ clinical levels of depression, stress and anxiety during the first week and the last week of lockdown showed that mental health outcomes changed over time and might be predicted by maladaptive personality traits (47). This recent literature suggests that lockdown can have a negative impact initially on psychological wellbeing, but also that some factors tend to stabilize over time. External factors, such as PA or daily activities, and internal factors such as personality and mindfulness traits, might predict the direction in which mental health outcomes develop. To the best of our knowledge, this is the first study investigating the evolution of how both internal and external variables influenced wellbeing over time.

### Behavioral Measures: Cognitive Tasks

One of the most used experimental paradigms to assess cognitive processes related to anxiety and stress is the Emotional Stroop Task [EST; (48)]. The EST effect involves a slower reaction time to threatening words compared to neutral ones, suggesting the allocation of attention toward threatening targets (an attentional bias). The task has been successfully used with individuals with panic disorder (49), post-traumatic stress disorder (50), generalized anxiety disorder (51), social phobia (52), and health anxiety (53).

When considering our experimental design, it is important to compare the EST to a task that assesses purely cognitive processes, not influenced by emotional or distressing features. Indeed, some studies have shown that COVID-19-related anxiety is associated with poorer cognitive performance (54, 55), as well as increased attention toward social cues (56). One of the most relevant and frequently used approaches to studying visual cognitive processing is through visual search tasks (57, 58). Performance efficiency in visual search tasks typically represents a reliable indicator of cognitive processing efficiency (59).

In the present longitudinal study, we invited participants to perform the EST (consisting of COVID-19 related and neutral words) and a visual search task every 7 days throughout the complete duration of the lockdown in Italy and the United Kingdom, to assess participants’ levels of anxiety toward the pandemic outbreak and their cognitive processing efficiency.

### Aims of the Research

In addition to examining the roles of potential protective and risk factors for mental health during the first lockdown in two European countries, this is the first study (to our knowledge) investigating the effects of lockdown and self-isolation on attention and vigilance. The aims were: (1) to characterize the prevalence of anxiety and depressive symptoms, and the overall levels of wellbeing, in an Italian and a United Kingdom sample at the start of lockdown; (2) to test whether anxiety, depression and wellbeing are correlated with personality traits and lifestyles at the beginning of the lockdown, since this may reveal coping strategies that can alleviate psychological distress and promote wellbeing at this critical time; (3) to examine whether anxiety, depression, and wellbeing are related to cognitive functioning at the beginning of the lockdown; (4) to understand how anxiety, depression, wellbeing and cognitive functioning evolve over the course of the lockdown; and (5) to explore how anxiety, depression and wellbeing are influenced by personality, mindfulness traits and daily habits over the course of the lockdown.

## Materials and Methods

### Participants

A sample of 96 participants was recruited to a 4-week longitudinal study. Twenty-five students from Kingston University (London, United Kingdom) signed up through an online research participation scheme in exchange for course credits. Seventy-one Italian participants were recruited *via* email or other social platforms (primarily Facebook). All sample characteristics (i.e., gender, age, job type, house size) are detailed in Table 1. Participants were informed of the study’s aims and they gave their electronic consent before starting the study. The research protocol was approved by the Kingston University Research Ethics Committee, and the study was conducted according to the ethical standards of the British Psychological Society and the Declaration of Helsinki 1964.

**TABLE 1:** Sample characteristics.

	Males	Females
Italy	*N* = 32	*N* = 39
	Age = 40 ± 14	Age = 41 ± 15
	Job type:	Job type:
	*Student* = *6*	*Student* = *7*
	*Occasional* = *3*	*Occasional* = *2*
	*Fixed-term* = *2*	*Fixed-term* = *5*
	*Permanent* = *10*	*Permanent* = *11*
	*Entrepreneur* = *9*	*Entrepreneur* = *8*
	*Retired* = *1*	*Retired* = *3*
	*Don’t know* = *0*	*Don’t know* = *2*
	*Unemployed* = *1*	*Unemployed* = *1*
	House size:	House size:
	<*40 mq/studio/1 bed* = *2*	< *40 mq/studio/1 bed* = *0*
	*40–80 mq/2 beds* = *8*	*40–80 mq/2 beds* = *10*
	*80–120 mq/3 beds* = *7*	*80–120 mq/3 beds* = *14*
	> *120 mq/* > *3 beds* = *15*	> *120 mq/* > *3 beds* = *15*
United Kingdom	*N* = 5	*N* = 20
	Age = 43 ± 17	Age = 23 ± 6
	Job type:	Job type:
	*Student* = *1*	*Student* = *16*
	*Occasional* = *0*	*Occasional* = *1*
	*Fixed-term* = *0*	*Fixed-term* = *0*
	*Permanent* = *3*	*Permanent* = *3*
	*Entrepreneur* = *0*	*Entrepreneur* = *0*
	*Retired* = *0*	*Retired* = *0*
	*Don’t know* = *0*	*Don’t know* = *0*
	*Unemployed* = *1*	*Unemployed* = *0*
	House size:	House size:
	<*40 mq/studio/1 bed* = *2*	<*40 mq/studio/1 bed* = *2*
	*40–80 mq/2 beds* = *0*	*40–80 mq/2 beds* = *5*
	*80–120 mq/3 beds* = *0*	*80–120 mq/3 beds* = *4*
	> *120 mq/* > *3 beds* = *3*	> *120 mq/* > *3 beds* = *9*

### Survey Overview

The web-based survey sent in the first week comprised 11 questionnaires. The first section focused on trait characteristics: demographic and general information about participants (i.e., age, gender, occupation, house size, household composition), personality traits (Big Five), pandemic impact as a traumatic personal event (IES-R), and the shorter version of the Five Facets Mindfulness Scale (FFMQ). The second part of the survey included a battery of questionnaires measuring state characteristics: questions about participants’ daily routines and habits, the shorter version of the SCL90-R (27 items), depression, anxiety and stress (DASS-21), sleep disorders (ISI) and general questions about drinking habits and nicotine/substance use. The survey was followed by two cognitive tasks assessing attention and vigilance. While personality and mindfulness traits, demographics and household information were asked only the first time, the second part of the survey assessing state characteristics and factors changing over time, was sent to participants during the remainder of the study (three subsequent sessions). On each session, behavioral data (attention and vigilance scores from the EST) were collected directly after participants finished the questionnaire (presented *via* Qualtrics); they were directed to the PsyToolkit web-based platform^1^ to perform the cognitive tasks. Participants were contacted by email every 7 days for 4 weeks and asked to complete the questionnaires and the cognitive tasks.

#### Questionnaires

We used the Big Five Inventory as a 44-items questionnaire measuring personality traits. This tool is based on the assumption that personality can be divided into five broad traits: Extraversion, Openness to Experience, Conscientiousness, Agreeableness, and Neuroticism (60). Items are answered on 5-point Likert scales ranging from 1: very inaccurate to 5: very accurate (i.e., “I see myself as curious about many different things”; “I see myself as worrying a lot”). The Italian version of the Big Five Inventory showed good internal reliability, with Cronbach’s alpha values ranging from 0.71 to 0.85 for the different subscales across three different samples (61).

The Depression, Anxiety and Stress Scale 21 (DASS-21; (62)) is a well-validated and effective tool that has widely been used to assess depression, anxiety and stress levels (divided into three factors) in clinical (63) and non-clinical studies (64), (i.e., “I found it hard to wind down”; “I found it difficult to work up the initiative to do things”). The DASS-21 has been shown to have good internal consistency: Cronbach’s alphas were 0.94 for Depression, 0.87 for Anxiety, and 0.91 for Stress (63).

The short version of the Insomnia Severity Index [ISI; (65)] was used to measure sleep difficulties. The shorter version has been validated and is highly correlated with the original version (66). The questionnaire assesses the severity of initial, middle and late insomnia, the variables being: sleep satisfaction, interference of insomnia with daytime functioning, noticeability of sleep problems by others, and distress about sleep difficulties (i.e., “How noticeable to others do you think your sleep problem is in terms of impairing the quality of your life?”; “How worried/distressed are you about your current sleep problem?”). ISI internal consistency was excellent for both clinical and non-clinical samples (alpha of 0.90 and 0.91) (67).

The Impact of Event Scale-Revised [IES-R; (68)] is a 22-item questionnaire to assess subjective responses to a specific traumatic cause (for this study, the instructions specify that the traumatic event is the outbreak of COVID-19 and its consequences). The tool consists of a set of affirmations measuring intrusion (intrusive thoughts, nightmares, intrusive feelings and imagery, dissociative-like re-experiencing), avoidance (numbing of responsiveness, avoidance of feelings, situations, and ideas), and hyperarousal (anger, irritability, hypervigilance, difficulty concentrating, heightened startle), (i.e., Instructions: “How much were you distressed or bothered by these difficulties?” Responses: “Any reminder brought back feelings about it”; “I avoided letting myself get upset when I thought about it or was reminded of it”). The IES-R demonstrated high internal consistency for the total scale (Cronbach’s alpha = 0.96), as well as for the three subscales (intrusion: 0.94; avoidance: 0.87; hyperarousal: 0.91) (69).

Mindfulness traits were assessed using the short form of the original Five Facet Mindfulness Questionnaire [FFMQ; (70)]. The FFMQ is a 39 items scale that measures five elements of mindfulness: observing, describing, acting with awareness, non-judging of and non-reactivity to the inner experience (i.e., “I’m good at finding words to describe my feelings”; “I notice how foods and drinks affect my thoughts, bodily sensations, and emotions”). The FFMQ-SF is a shorter version consisting of 24 items and has been validated as a measure of the variables related to mindfulness (71). The Italian version of the FFMQ showed good to excellent internal consistency as a whole (alpha = 0.86) with sub-scale consistency ranging from 0.65 to 0.81 (72).

The Symptoms Checklist Revised (SCL-90-R) is widely used to assess a range of psychopathological symptoms (27). The short form of the questionnaire, which comprises 27 of the original 90 items [SCL-27; (73)], was used here. The symptoms can be categorized into six subscales: depressive symptoms, dysthymia symptoms, vegetative symptoms, agoraphobic symptoms, symptoms of social phobia and symptoms of mistrust. In our analyses, the total score was the main measure (i.e., Instructions: “For the past week, how much were you bothered by:” Responses: “Trouble remembering things”; “Feeling low in energy or slowed down”). All scales of the SCL-27 showed good to satisfactory reliability (i.e., Cronbach’s alpha between 0.70 and 0.90) (73).

#### Daily Habits

A list of 29 activities (i.e., web browsing, reading newspaper, painting etc.) was used to assess participants’ daily routines and “going outside” habits. Items were answered on 5-point Likert scales ranging from 1: I do not carry out this activity to 5: More than 3 h a day. The complete questionnaires are shown in the Supplementary Material.

#### Cognitive Tasks

Two cognitive tasks were used: the EST and the Visual Search Task. The EST is widely used in both clinical and basic research (48). The task measures the impact of emotional stimuli on attentional processes, with prolonged response latencies recorded when participants name the ink colors of emotional words compared to neutral words, indicating so-called emotional interference (measured as mean response latency for emotional words minus mean response latency for neutral words). Many studies have examined attentional biases for trauma-related stimuli using the EST [e.g., (74)]. The EST is based on the assumption that attentional biases are driven by bottom-up processing: attention is involuntary and automatically directed toward threatening stimuli, and might impair participants’ performance [e.g., (48, 49)]. In this study, the EST consisted of 25 neutral and 25 coronavirus-related (e.g., pandemic, isolation, infection) sets of words, repeated twice (100 trials in total).

The visual search paradigm is a task in which participants seek a specific target item among several non-targets (distractors) and they are asked to press a key if the target is present. The task measures visual perception and selective attention based on the Feature-Integration Theory of perception (75). According to this theory, the visual search process consists of two sequential stages: (i) the first stage is early, preattentive and perceptive, consisting of a fast parallel search of a single target feature; (ii) the second stage is late, attentive and consists of a slower serial search of all objects in the visual scene, aimed at identifying specific conjunction of more than one target features. The time needed to identify the target item increases as the number of distractors increases in the second stage (i.e., serial search, when looking for specific conjunction of features). This task is aimed at evaluating participants’ visual processing skills, independently of semantically relevant information (which is manipulated in the EST task). A large number of studies have validated the visual search paradigm to explore visual attention mechanisms in healthy and clinical populations [e.g., (76–79)]. The task included 50 trials in total, i.e., 25 with the target and 25 without the target, presented in random order. On each trial, participants were asked to press the spacebar if they found the target figure (e.g., a red T among inverted Ts and blue Ts, acting as distractors). The set size (i.e., the number of distractors + target) was randomly determined among 5, 10, 15, and 20 figures in each trial. Feedback was given after each trial if the participant’s response was incorrect.

### Statistical Analyses

#### Main Analyses

All analyses were guided by five research questions based on the five research aims stated in the Introduction:

• Q1. What is the prevalence of anxiety and depressive symptoms, and the overall levels of wellbeing, in an Italian and a United Kingdom sample at the start of lockdown?
• Q2. Are anxiety, depression and wellbeing correlated with personality traits and lifestyle at the beginning of the lockdown?
• Q3. Are anxiety, depression, and wellbeing related to cognitive functioning at the beginning of the lockdown?.
• Q4. How does the relationship between anxiety, depression, wellbeing and cognitive functioning evolve over the course of the lockdown?
• Q5. How are anxiety, depression and wellbeing influenced by personality, mindfulness traits and daily habits over the course of the lockdown?

To address the first research question (Q1), we explored mean values of the wellbeing variables (i.e., depression, anxiety, stress in DASS-21, insomnia in ISI, SCL-27 total score) for the first week of data collection (T1) and compared the mean values with the normative sample mean of each questionnaire using a *t*-test to investigate whether our sample was statistically different from the general population. We also examined how many participants showed an IES-R total score above the clinical threshold [thus suggesting a possible post-traumatic stress disorder diagnosis; threshold = 33, (80)]. The same was done for the ISI insomnia score [clinical threshold = 14, (67)].

For Q2, at the first time point (T1) we tested the relationship between both internal (i.e., Big Five personality traits and FFMQ mindfulness traits) and external (i.e., house characteristics, daily routines components, “going outside” habits components, and use of nicotine) independent variables and wellbeing dependent variables (i.e., depression, anxiety, stress in DASS-21, and SCL-27 total score) in two multivariate linear models: the first model included the three dependent variables from DASS-21 in a multivariate design (since they are correlated by definition), while the second model tested the effects on the SCL-27 total score separately. Data from the substance use questions were excluded from these analyses as a preliminary analysis found very sparse data (the vast majority of participants reported no substance use), perhaps affected by a social desirability bias (81). In all models, days after the lockdown began were added as an independent variable. By using the exact number of days after the beginning of lockdown, we covaried the differences related to the specific dates that participants reported as lockdown start. In these models, the statistical significance threshold was lowered to α = 0.0125 to avoid type I errors (false positives); since we tested four different measurements in time, we divided the α value by four as in a typical Bonferroni correction for multiple comparisons.

To address Q3, we tested the correlation matrix between participants’ performance on each cognitive task (i.e., EST and Visual Search Task) and the same wellbeing variables cited above for T1. To compute participants’ performance on the tasks, Inverse Efficiency Scores (IES) were calculated as the ratio between individual response times (RTs after removing outlier scores more than 2 SD from the mean, computed for each participant separately) and the mean proportion of correct answers. For the EST, an emotional interference index was computed as the difference between individual IES in trials related to coronavirus and IES in neutral trials. Therefore, the higher this index, the greater the interference created by coronavirus-related trials (i.e., worse performance–higher IES–in coronavirus-related trials than in neutral trials). For the Visual Search task, a serial visual search cost index was computed as the difference between individual IES in trials with the highest set size (i.e., number of figures in the display set; 20) and the lowest set size (5). Therefore, the higher this index, the greater was the cost of serial visual search in terms of performance decrease. To test the evidence in favor of null hypothesis (H_0_) vs. alternative hypothesis (H_1_), we also computed a Bayesian correlation matrix, displaying the Bayes Factor for each correlation. In this case, the Bayes Factor (BF_01_) reflects the ratio between the likelihood of the data given H_0_ and the likelihood of the data given H_1_ (82). In other words, the higher the BF, the more likely are the data given one of the two hypotheses.

Analyses related to Q4 were extensions of the previous analysis, as we tested the same correlation matrix, but used the angular coefficients of these variables over time. The angular coefficient was computed by fitting a regression for each participant for each score (dependent variable) and using the days after the lockdown start as the independent variable. Therefore, these coefficients represented the variation of these variables during the lockdown, in other words, the individual increase or decrease over time. As with Q2, by using the exact number of days after the beginning of lockdown, we covaried the differences related to the specific dates that participants reported as lockdown start. Since we needed at least two time points to fit a regression line for each participant, only participants with at least two recordings of all variables were included in this analysis. Indeed, participants who failed to complete the survey after the second week (*N* = 27) were excluded from the longitudinal analysis, yielding a final sample of 69 participants for Q4 and Q5 analyses. Again, a Bayesian correlation matrix was computed to display the evidence for H_0_ vs. H_1_.

In the same fashion, the analyses for Q5 were effectively the same as for Q2 but extended longitudinally. Data from all questionnaires were analyzed cross-sectionally, thus testing how the relationships among variables changed over time. Therefore, separate models were created for each week of data collection, from T1 (first week) to T4 (final week). Each model investigated the relationship between the same independent and dependent variables described in Q2. The α = 0.0125 threshold was used also in these models (as in Q2), to avoid type I errors.

All statistical analyses were performed using RStudio software (83), and the following packages: Lavaan (84), Psych (85), and Dplyr (86).

#### Principal Components Analyses

In order to reduce dimensionality and to identify commonalities in data from the two questionnaires about daily routines and “going outside” habits, we performed two Principal Components Analyses (PCAs) on these data (87). The criteria used to choose the number of components were (1) scree plot, (2) eigenvalue of each component >1, (3) results interpretability. Tables for each criterion are reported for the two PCAs in the Results section. Factors rotation was chosen based on the correlation between components, i.e., oblique rotation (“*oblimin*”) when at least one correlation was >0.2 or <−0.2; orthogonal rotation (“*varimax*”) when no correlations showed values above or below the aforementioned thresholds. Factorial scores from the two PCAs were saved and used as individual scores in further analyses.

## Results

### Principal Components Analyses

The PCA performed on daily routines highlighted a 5-component solution, with “*varimax*” rotation, as the highest correlation among components in a solution with oblique rotation was 0.19. Loadings and explained variance are given in Table 2. In the 5-component solution that we used, contemplative and experiential habits loaded on component 1 (practicing yoga/pilates, practicing mindfulness, listening to podcasts, painting, cooking, listening to audiobooks); TV-related habits loaded on component 2 (watching TV, watching movies or series, and negatively playing musical instruments, and reading); social and non-social activities loaded on component 3 (listening to the radio, calling friends, and negatively watching Youtube); internet-related and physical activities loaded on component 4 (watching Youtube, using social networks, listening to music, web browsing, and doing PA); recreational activities loaded on component 5 (playing card games, attending online courses, playing videogames, watching the news).

**TABLE 2:** PCA loadings for daily routines.

	Loading cutoff = 0.40				
Routine	RC1	RC5	RC4	RC2	RC3
Practicing yoga/pilates	**0.73**	−0.18	−0.15	−0.10	0.06
Practicing mindfulness	**0.64**	0.14	0.22	−0.31	−0.14
Listening to podcasts	**0.55**	0.25	0.29	0.03	0.10
Painting	**0.66**	0.06	0.09	−0.01	0.29
Cooking	**0.56**	0.02	−0.37	0.12	0.01
Listening to audiobooks	**0.61**	0.36	0.17	−0.02	0.07
Playing card games	0.15	**0.66**	0.08	0.00	0.09
Attending online courses	0.16	**0.59**	0.00	−0.04	0.09
Playing videogames	−0.05	**0.65**	0.26	0.10	−0.30
Watching Youtube	0.32	0.20	**0.55**	−0.02	−**0.47**
Using social networks	−0.04	−0.14	**0.68**	0.12	0.37
Listening to music	0.05	0.11	**0.65**	−0.29	0.12
Watching movies or series	0.09	0.19	0.17	**0.65**	0.00
Watching TV	0.04	0.18	−0.01	**0.61**	0.37
Playing musical instruments	0.26	0.35	0.15	−**0.58**	0.06
Listening to the radio	0.09	0.29	−0.02	0.00	**0.55**
Calling friends	0.19	−0.06	0.15	−0.01	**0.58**
Web browsing	−0.02	0.14	**0.41**	0.36	−0.10
Doing physical activity	0.05	0.05	**0.45**	0.05	−0.02
Watching the news	−0.24	**0.47**	−0.17	0.27	0.25
Reading	0.21	0.21	0.10	−**0.46**	0.28
**RC = Rotated component**					
	**RC1**	**RC5**	**RC4**	**RC2**	**RC3**
**SS loadings**	2.73	2.10	2.07	1.77	1.53
**Proportion variance**	0.13	0.10	0.10	0.08	0.07
**Cumulative variance**	0.13	0.23	0.33	0.41	0.49

The PCA performed on “going outside” habits produced a 4-component solution, with “*varimax*” rotation, as the highest correlation among components in a solution with oblique rotation was 0.14. Loadings and explained variance are given in Table 3. In the 4-component solution we used, indispensable activities loaded on component 1 (working, and going to the off-license/tobacco shop); buying groceries, going to the pharmacy, and buying newspapers loaded on component 2; going outside for PA loaded on component 3; going outside to walk the dog loaded on component 4.

**TABLE 3:** PCA loadings for “going outside” habits.

	Loading cutoff = 0.40			
Habit	RC1	RC2	RC3	RC4
Working	**0.80**	0.15	0.11	−0.17
Going to the off license/tobacco shop	**0.84**	−0.08	−0.17	0.19
Buying groceries	0.36	**0.61**	0.33	−0.33
Going to the pharmacy	−0.03	**0.77**	0.07	0.09
Buying newspapers	0.03	**0.64**	−0.38	0.14
Physical activity	−0.04	−0.01	**0.91**	0.11
Walking the dog	0.02	0.11	0.10	**0.94**
**RC = Rotated component**				
	**RC1**	**RC2**	**RC3**	**RC4**
SS loadings	1.48	1.41	1.13	1.10
Proportion variance	0.21	0.20	0.16	0.16
Cumulative variance	0.21	0.41	0.57	0.73

### Q1: Prevalence of Anxiety and Depressive Symptoms

Descriptive statistics for all main variables are shown in Table 4. The *t*-tests against normative samples scores indicated significantly higher scores in our sample compared to the general population for the DASS: depression [participants’ mean = 11.57; validation mean = 7.19; *t*(91) = 4.36, *p* < 0.001], stress [participants’ mean = 13.72; validation mean = 10.54; *t*(91) = 3.06, *p* = 0.003]; SCL-27 total score [participants’ mean = 0.75; validation mean = 0.52; *t*(95) = 3.87, *p* < 0.001]. However, the DASS anxiety score did not differ significantly [participants’ mean = 6.09; validation mean = 5.23; *t*(91) = 0.996, *p* = 0.322]. IES-R scores revealed that 21% of participants (20/96) displayed a possible post-traumatic stress disorder diagnosis (total score >33) in T1, while only 3% of participants (3/96) showed a diagnostic value in the ISI (score >14). In summary, several indices showed increased levels of depression, stress, psychiatric symptoms and risk of PTSD in participants in lockdown.

**TABLE 4:** Descriptive statistics in T1.

	IT (71)		United Kingdom (25)	
Variable	M (32)	F (39)	M (4)	F (20)
DASS: Depression	6.94 (5.88)	11.89 (9.65)	14.0 (19.80)	17.60 (10.50)
DASS: Anxiety	1.63 (2.18)	5.62 (7.30)	7.00 (9.90)	13.60 (10.75)
DASS: Stress	8.56 (7.19)	15.62 (9.66)	9.00 (12.73)	19.00 (11.08)
ISI: Insomnia	5.28 (4.42)	6.86 (4.80)	1.00 (1.51)	3.50 (0.71)
SCL-27: Total score	0.40 (0.29)	0.77 (0.51)	0.61 (0.61)	1.14 (0.70)

*DASS, depression anxiety stress scale–21 items version; ISI, insomnia severity index; SCL-27, symptom checklist–27 items version.*

### Q2: How Anxiety, Depression and Wellbeing Are Correlated With Personality, Mindfulness and Lifestyle at the Beginning of the Lockdown

All of the results from these models are detailed in the T1 section in the Supplementary Material. Protective effects were statistically negative (since depression/anxiety/stress/symptoms scores decreased as the variable of interest increased), while detrimental effects were statistically positive (since depression/anxiety/stress/symptoms scores increased as the variable of interest increased). Only internal variables showed statistically significant effects on wellbeing variables in T1: we found significant protective effects of openness (on depression: *b* = −0.891, *z* = −4.263, *p* < 0.001; on anxiety: *b* = −0.488, *z* = −2.749, *p* = 0.006; on stress: *b* = −0.858, *z* = −3.765, *p* < 0.001; on SCL-27: *b* = −0.039, *z* = −3.206, *p* = 0.001) and detrimental effects of neuroticism (on depression: *b* = 0.706, *z* = 3.182, *p* = 0.001; on anxiety: *b* = 0.518, *z* = 2.747, *p* = 0.006; on stress: *b* = 0.959, *z* = 3.965, *p* < 0.001; on SCL-27: *b* = 0.051, *z* = 4.242, *p* < 0.001) on all four dependent variables (i.e., DASS: depression, anxiety, stress; SCL-27 total score). Moreover, we found a significant detrimental effect of the FFMQ factor “description” on DASS: depression (*b* = 1.082, *z* = 2.656, *p* = 0.008) and a significant protective effect of conscientiousness on the DASS component stress (*b* = −0.484, *z* = −2.737, *p* = 0.006). In summary, only internal variables showed an effect on wellbeing at the beginning of the lockdown. In particular, openness and conscientiousness had protective value, while neuroticism and description had a detrimental effect.

### Q3: How Anxiety, Depression, and Wellbeing Are Related to Cognitive Functioning at the Beginning of the Lockdown

The frequentist and Bayesian correlation matrices for T1 between behavioral tasks scores and wellbeing scores are given in Table 5. The only statistically significant negative correlation was between the Visual Search cost index and the DASS: stress score. Additionally, the Bayesian correlations showed general evidence for the null hypothesis, with BF_01_ ranging from 1.977 to 5.868. These results suggest that the data are 1.977 to 5.868 more likely to be observed under H_0_ than under H_1_. Bayesian correlation indicated an inconclusive outcome concerning the only statistically significant correlation (between the Visual Search cost index and the DASS: stress score), with a BF_01_ = 0.775. This Bayes Factor value does not consistently support either H_0_ or H_1_.

**TABLE 5:** Frequentist and Bayesian correlation matrix in T1 between behavioral tasks scores and wellbeing variables.

	DASS: Depression	DASS: Anxiety	DASS: Stress	SCL-27: Total score
Emotional stroop task: Emotional interference index	*r* = 0.10	*r* = 0.12	*r* = 0.06	*r* = 0.12
	*p* = 0.42	*p* = 0.36	*p* = 0.66	*p* = 0.33
	BF_01_ = 4.72	BF_01_ = 4.26	BF_01_ = 5.87	BF_01_ = 4.07
Visual search task: Serial visual search cost index	*r* = −0.16	*r* = −0.13	*r* = −0.26	*r* = −0.19
	*p* = 0.20	*p* = 0.29	*p* = 0.04*	*p* = 0.12
	BF_01_ = 2.91	BF_01_ = 3.72	BF_01_ = 0.775	BF_01_ = 1.98

DASS, depression anxiety stress scale–21 items version; ISI, insomnia severity index; SCL-27, symptom checklist–27 items version. *Statistically significant effect, p < 0.05.

### Q4: How the Relationship Between Anxiety, Depression, Wellbeing and Cognitive Functioning Evolves Over the Course of the Lockdown

The frequentist and Bayesian correlation matrices between the time course of behavioral tasks scores and wellbeing scores are detailed in Table 6. No statistically significant effects were identified between the variables of interest. Bayesian correlations suggested only anecdotal-to-medium support for H_0_ (BF_01_ ranging from 2.427 to 5.579). In summary, wellbeing did not show any significant correlations with cognitive functioning, neither at the beginning of the lockdown (Q3) nor over its course (Q4).

**TABLE 6:** Frequentist and Bayesian correlation matrix between angular coefficients of behavioral tasks scores and wellbeing variables over time.

	DASS: Depression	DASS: Anxiety	DASS: Stress	SCL-27: Total score
Emotional stroop task: Emotional interference index	*r* = 0.03	*r* = −0.19	*r* = 0.09	*r* = −0.03
	*p* = 0.82	*p* = 0.19	*p* = 0.53	*p* = 0.86
	BF_01_ = 5.52	BF_01_ = 2.43	BF_01_ = 4.69	BF_01_ = 5.58
Visual search task: Serial visual search cost index	*r* = −0.08	*r* = −0.14	*r* = 0.06	*r* = −0.11
	*p* = 0.58	*p* = 0.33	*p* = 0.68	*p* = 0.46
	BF_01_ = 4.89	BF_01_ = 3.54	BF_01_ = 5.23	BF_01_ = 4.36

DASS, depression anxiety stress scale–21 items version; ISI, insomnia severity index; SCL-27, symptom checklist–27 items version. *Statistically significant effect, p < 0.05.

### Q5: How Anxiety, Depression and Wellbeing Are Influenced by Personality, Mindfulness and Lifestyle Over the Course of the Lockdown

All cross-sectional results from these models are detailed in the Supplementary Material. A summary of the main findings from cross-sectional analyses is also shown in Table 7. Results for T1 are given above (Q2). For T2, we found statistically significant protective (statistically negative) effects of the FFMQ factor “non-judgment” on DASS factor depression (*b* = −1.290, *z* = −2.503, *p* = 0.012), an effect of presence of balcony on DASS factor anxiety (*b* = −4.345, *z* = −2.708, *p* = 0.007) and an effect of openness on the SCL-27 total score (*b* = −0.036, *z* = −2.629, *p* = 0.009). The protective effect of “non-judgment” on DASS factor depression remained significant also in T3 (*b* = −1.552, *z* = −2.713, *p* = 0.007), while no other statistically significant outcomes were apparent at this time point. For T4, only external variables showed significant effects on wellbeing: daily routines component 3 (social and non-social activities) produced a detrimental (statistically positive) effect on DASS: depression (*b* = 5.135, *z* = 3.075, *p* = 0.002) and the SCL-27 total score (*b* = 0.333, *z* = 4.416, *p* < 0.001). Also, the household variable (i.e., number of households) showed a detrimental effect on the SCL-27 total score (*b* = 0.187, *z* = 3.768, *p* < 0.001), while daily routines component 2 (TV- and internet-related habits; *b* = −0.253, *z* = −3.225, *p* = 0.001), “going outside” component 4 (walking the dog; *b* = −0.237, *z* = −3.042, *p* = 0.002) and presence of balcony (*b* = −0.562, *z* = −2.648, *p* = 0.008) showed a protective (negative) effect on the SCL-27 total score. In summary, over the course of the lockdown, internal variables (personality and mindfulness scales) gradually lost relevance as predictors of wellbeing, while external variables (house characteristics and lifestyle) increased their predictive power by the end of the lockdown.

**TABLE 7:** Summary of the main findings from cross-sectional analyses.

TIME	DASS: Depression	DASS: Anxiety	DASS: Stress	SCL-27: Total score
T1	BF: NEUROTICISM	BF: NEUROTICISM	BF: NEUROTICISM	BF: NEUROTICISM
	BF: OPENNESS	BF: OPENNESS	BF: OPENNESS	BF: OPENNESS
	FFMQ: DESCRIPTION		BF: CONSCIENTIOUSNESS	
T2	FFMQ: NON-JUDGMENT	BALCONY		BF: OPENNESS
T3	FFMQ: NON-JUDGMENT			
T4	HABIT: COMPONENT 3 (social and non-social activities)			HOUSEHOLD
				HABIT: COMPONENT 2 (TV-related habits)
				HABIT: COMPONENT 3 (social and non-social activities)
				GOING OUTSIDE: COMPONENT 4 (walk the dog)
				BALCONY

*GREEN VARIABLES = significant protective (statistically negative) effects.*

*RED VARIABLES = significant detrimental (statistically positive) effects.*

*DASS, depression anxiety stress scale–21 items version; ISI, insomnia severity index; SCL-27, symptom checklist–27 items version; BF, big five questionnaire; FFMQ, five factors mindfulness questionnaire.*

## Discussion

Lockdown and self-isolation are measures deployed during the COVID-19 pandemic that have been shown to negatively affect individuals in different ways. However, several resilience factors can help individuals to cope better with self-isolation. This study aimed to identify resilience factors that could mitigate the negative impacts of the COVID-19 quarantine on mental health through a longitudinal methodology that evaluated participants during the first lockdown in Italy and the United Kingdom.

Overall, our study showed that self-reported measures of psychological distress were elevated relative to average scores for the general adult population. Participants reported higher levels of depression and stress during the COVID-19 lockdown, which is consistent with results from other studies during the same period [e.g., (88–91)]. The sample reported higher scores on the SCL-27 compared with population norms, supporting recent studies that have indicated an increase in symptom scores measured by the SCL-27 during self-isolation (92). Potential internal and external factors that played a role in self-isolation were also investigated including personality and mindfulness traits, daily routines, and living conditions. Indeed, longitudinal analyses revealed that internal variables influenced participants’ wellbeing more during the first weeks of the study, while external variables influenced it more during the last measurements.

### Internal Factors: Personality and Mindfulness Traits

Two personality traits, neuroticism and openness, affected DASS subscales in the first week, suggesting that those who score high for these traits are more likely to experience greater distress, anxiety, and depression. Previous findings from longitudinal studies have also shown that neuroticism can negatively affect mental health outcomes (47). However, these effects seem to be stable and do not increase over time, indicating an ability to adapt to the negative consequences of lockdown after 1 week. In accordance with the present results, previous longitudinal studies conducted in different countries have demonstrated changes in wellbeing indicators during different stages of lockdown (10, 46, 93). Ruggieri et al. (46) conducted a 4-week study between the 7th March and 14th April 2020 measuring loneliness, anxiety, depression, and life satisfaction among an Italian sample. Similar findings were reported from longitudinal studies conducted in China (10) and in the United States (93). Consistent with the literature, our research suggested significant deleterious effects after the first week, followed by stabilization of the scores during the subsequent quarantine period, supporting the view that individuals may adapt to the negative consequences of social isolation and home confinement (10, 46, 93).

This study also investigated the contribution of mindfulness facets to depression, anxiety, and stress during the lockdown. The ability to describe feelings, thoughts, and experiences with words seemed to be a predictor for depression in T1. This could be explained by the fact that high introspection scores may have reflected excessive rumination. It could be that distraction, although less efficient in the long run, could have been a better emotion regulation strategy in the specific context of the pandemic. Self-reported scores were collected at the beginning of a pandemic, when people knew very little about it and were required to deal emotionally with a completely novel situation based on uncertainty. In contrast, having a non-judgmental attitude predicted lower levels of depression at T2 and T3. Our findings are in line with the results from Medvedev et al. (94), which suggested that having a non-judgmental attitude could protect against depression over time. Together, these outcomes demonstrate that several aspects of mindfulness (in particular not being judgmental) might protect against depression in both normal and emergency conditions, especially during social isolation. Indeed, a systematic meta-analysis (95) found a significant increase in mindfulness and lower levels of psychological stress in participants using mindfulness apps. A recent study has also shown that an online mindfulness intervention significantly reduced perceived stress in Singaporean participants during lockdown (96), and online mindfulness training has also been shown to reduce anxiety and depression in people suffering from COVID-19 during isolation (97).

### External Factors

#### Daily Routines: Digital Activities

Self-isolation and lockdown often forced individuals to engage in social-digital interactions [e.g., video calls] and to spend more time using social media platforms. Our results showed that an increase in time spent using social media and engaging in video calls had detrimental effects on individuals during the last (fourth) week of the study, thus suggesting a negative influence of digital activities if used over time. This finding is contrary to previous results which have suggested that people benefit from an increase in digital activities to compensate for loneliness (42, 43). Kopilaš et al., (42) looked at the role of digital activity during self-isolation in Croatia and Italy and suggested that participants increased digital activity to provide socialization when physically distancing. Their findings showed that digital activities, such as social media activity and the use of computers and smartphones, were associated with higher scores on the PANAS positive affect scale, supporting the assumption that digital activities might serve as a protective factor during self-isolation. A further longitudinal study investigating the role of social media and video calls during the lockdown has noted the importance of digital interactions and their positive consequences on participants’ self-report depression scores (43). However, our findings support the suggestion by Islam et al. (41) that a negative effect of social media can occur due to the overwhelming amount of information that can be associated with it. Recent literature defined the excessive use of social media during the pandemic as a technological/social paradox, in which individuals increased their use of the internet to stay in contact with family and friends, due to the government’s restrictions on social contact; nevertheless, the ubiquity of this medium in our life is also a cause of an increase of techno-stress (98, 99). Moreover, our sample consists of people of different ages and middle-aged or older individuals who may find communication using social media less beneficial, if not frustrating in the current circumstances.

The current study also showed that some digital activities can have positive consequences on individuals: watching television, movies, and series were associated with positive effects, and therefore were protective factors. However, this was not the case in a recent study that reported an association between depression and increased time spent watching television, movies, and playing computer games (100).

#### Daily Routines: Walking the Dog

Participants that engaged in dog walking reported fewer negative symptoms during the last week of the study, supporting the assumption that pets can prove beneficial during lockdowns (37, 101, 102). Ratschen et al. (103) also found that individuals who owned a pet showed less deterioration in mental health and reduced loneliness. Walking a dog also necessitates PA; Moore et al. (104) in Canada reported a positive correlation between family dog ownership and increased PA. Dog ownership and dog walking can therefore be considered protective factors during lockdown (37–39).

#### House Features

Different house features can also affect individuals’ self-reported wellbeing during self-isolation. The results support previous findings suggesting that the presence of a balcony or a patio can contribute toward coping better with the lockdown situation (105–107) since private outdoor access (balcony) was associated with lower levels of anxiety during part of the lockdown (second week of the study). In contrast, the larger the number of people in the household the higher the SCL scores reported, suggesting that people were more likely to be dissatisfied with their housemate relationships over time, as reflected in the outcome during the last week of the study.

### Cognitive Functioning

The cognitive measures showed no statistically significant correlations with wellbeing indices, and Bayesian tests consistently indicated a lack of evidence for any correlations. Considering the EST, these results seem to suggest that the anxiety and stress related to COVID-19 are efficiently captured by explicit measures (i.e., self-report questionnaires) but the lockdown did not particularly influence the attention toward emotional material. The Visual Search Task showed that selective attention, a purely cognitive function, was not affected by the psychological state during the lockdown. Therefore, our results suggest that the pandemic did not have an impact on attentional mechanisms, relating either to “emotional” or “non-emotional” stimuli. Previous literature showed that the COVID-19 lockdown was associated with poorer cognitive performance (54, 55) and increased attention toward social cues (56). Our findings seem to go against these results; this discrepancy likely reflects the specific components investigated and the measures used, since previous studies primarily focused on working memory (especially using n-back tasks) or gaze cueing tasks. Alternatively, these behavioral measures may have been affected by certain limitations: (i) online cognitive tasks were completed by fewer participants compared to the surveys; (ii) in some cases the cognitive tasks were completed at a different time from the surveys, making these data difficult to align or compare; (iii) online behavioral tasks cannot be as rigorously controlled as in-person laboratory experiments. Therefore the absence of significant correlations between behavioral tasks and wellbeing indices might reflect these limitations rather than the lack of any influence.

### Limitations and Future Directions

This study has a number of strengths and limitations. While our results allow crucial insights on how internal and external variables influence wellbeing at different phases of a social lockdown, the main limitation is related to the sample size over time. The number of participants completing the weekly surveys decreased over time (as shown by the lower number of participants included in longitudinal analyses), mainly due to lack of engagement. This was probably related to the absence of monetary reward or other incentives, but this allowed us to avoid a selection bias toward participants who were reward-driven. The small sample size and the relevant attrition rate (as well as the oversampling of the active population, i.e., 15 to 64 years) cannot allow us to generalize our results to the general population, especially outside the countries we studied. Given the small sample size and in order to test the statistical power of our models, we ran an *a posteriori* power analysis which supported the statistical reliability of our results (see the Supplementary Material for the complete analysis). Further research is needed to explore cognitive variables (i.e., attention) that might be affected during stressful situations and in future lockdowns, possibly using more controlled settings. Another possible confound in this study is represented by the possible issues in interpreting the results from the IES-R questionnaire as a pure index of the pandemic traumatic impact. As a matter of fact, this scale investigated generic post-traumatic symptoms (with no specific reference to the lockdown) and, therefore, any other traumatic event may have implications on our results. Nevertheless, the pandemic may have represented the main traumatic event for most participants, given the overwhelming change in routines they experienced.

The implications of this study could be important, as it has provided new insights about how to address different phases of a social lockdown brought on by a national emergency. Health services may be better able to identify the most susceptible people (e.g., in terms of personality structure, during early phases; persons in difficult housing conditions in later phases) and help prevent a worsening of their psychological state by creating targeted interventions. Another important aspect to consider in future research is testing how these effects on wellbeing change over longer timeframes, e.g., after the end of the first wave lockdown and during subsequent waves of COVID-19 with consequent restrictions.

## Conclusion

Self-isolation may have important consequences on individuals over time. The current study showed that internal factors, such as personality and mindfulness traits, were more predictive for wellbeing during the first weeks, but stabilized over time. In contrast, external factors, such as daily routines and house features, predicted more strongly a person’s psychological wellbeing during the last weeks of the study.

This is the first study to our knowledge that adopted a mixed-methods approach to assess changes in psychological wellbeing and attention over time. Although our behavioral findings did not show any association between cognitive performance and psychological wellbeing, this aspect deserves further study in the future. Our findings contribute to the growing literature that supports the use of tailored interventions for individuals who may struggle during self-isolation. Educational interventions implemented within the workplace or educational settings could guide individuals to learn protective and coping strategies that might prevent the negative effects of self-isolation.

## Data Availability Statement

The raw data supporting the conclusions of this article will be made available by the authors, without undue reservation.

## Ethics Statement

All participants were provided with an exhaustive description of all the experimental procedures and were required to sign a written informed consent before taking part in the study. The study was conducted in accordance with the ethical standards laid down in the 1964 Declaration of Helsinki and under a protocol approved by the Kingston University Ethics Committee (protocol #2020-1549).

## Author Contributions

FZ, FB, and GG contributed to the design and the conception of the research. FZ and FB contributed to the implementation, administration of the surveys and behavioral tasks, and contributed to writing of the manuscript. FB and MR contributed to the analysis of the results. All authors contributed to the manuscript revision, read, and approved the submitted version.

## Conflict of Interest

The authors declare that the research was conducted in the absence of any commercial or financial relationships that could be construed as a potential conflict of interest.

## Publisher’s Note

All claims expressed in this article are solely those of the authors and do not necessarily represent those of their affiliated organizations, or those of the publisher, the editors and the reviewers. Any product that may be evaluated in this article, or claim that may be made by its manufacturer, is not guaranteed or endorsed by the publisher.

## Footnotes

1
http://psytoolkit.org/

## References

[1] World Health Organization. *Considerations for Quarantine Of Individuals In The Context Of Containment For Coronavirus Disease (ıı COVID-19)ıı: Interim Guidance, 19 March 2020 (No. WHO/2019-nCoV/IHR_Quarantine/2020.2).* Geneva: World Health Organization (2020).

[2] Ministero della Salute. *Decreto del Presidente del Consiglio dei Ministri 11 Marzo 2020 [Decree of the Prime Minister 11 March 2020]*. (2020).

[3] Jaspal R Nerlich B. Social representations, identity threat, and coping amid COVID-19. *Psychol Trauma.* (2020) 12:S249. 10.1037/tra0000773 32463288

[4] Ahmed MZ Ahmed O Aibao Z Hanbin S Siyu L Ahmad A. Epidemic of COVID-19 in China and associated psychological problems. *Asian J Psychiatry.* (2020) 51:102092. 10.1016/j.ajp.2020.102092 32315963 PMC7194662

[5] Brooks SK Webster RK Smith LE Woodland L Wessely S Greenberg N The sychological impact of quarantine and how to reduce it: rapid review of the evidence. *Lancet.* (2020) 395:912–20. 32112714 10.1016/S0140-6736(20)30460-8 PMC7158942

[6] Casagrande M Favieri F Tambelli R Forte G. The enemy who sealed the world: effects quarantine due to the COVID-19 on sleep quality, anxiety, and psychological distress in the Italian population. *Sleep Med.* (2020) 75:12–20. 10.1016/j.sleep.2020.05.011 32853913 PMC7215153

[7] Ko NY Lu WH Chen YL Li DJ Wang PW Hsu ST COVID-19-related information sources and psychological well-being: an online survey study in Taiwan. *Brain Behav Immun.* (2020) 87:153. 10.1016/j.bbi.2020.05.019 32389702 PMC7204755

[8] Moccia L Janiri D Pepe M Dattoli L Molinaro M De Martin V Affective temperament, attachment style, and the psychological impact of the COVID-19 outbreak: an early report on the Italian general population. *Brain Behav Immun.* (2020) 87:75–9. 10.1016/j.bbi.2020.04.048 32325098 PMC7169930

[9] Tian H Liu Y Li Y Wu CH Chen B Kraemer MU An investigation of transmission control measures during the first 50 days of the COVID-19 epidemic in China. *Science.* (2020) 368:638–42. 10.1126/science.abb6105 32234804 PMC7164389

[10] Wang C Pan R Wan X Tan Y Xu L McIntyre RS A longitudinal study on the mental health of general population during the COVID-19 epidemic in China. *Brain Behav Immun.* (2020) 87:40–8. 32298802 10.1016/j.bbi.2020.04.028 PMC7153528

[11] Southwick SM Sippel L Krystal J Charney D Mayes L Pietrzak R. Why are some individuals more resilient than others: the role of social support. *World Psychiatry.* (2016) 15:77. 26833614 10.1002/wps.20282 PMC4780285

[12] Caspi A Moffitt TE. When do individual differences matter? A paradoxical theory of personality coherence. *Psychol Inq.* (1993) 4:247–71.

[13] Kotov R Gamez W Schmidt F Watson D. Linking “big” personality traits to anxiety, depressive, and substance use disorders: a meta-analysis. *Psychol Bull.* (2010) 136:768. 10.1037/a0020327 20804236

[14] Qian GQ Yang NB Ding F Ma AHY Wang ZY Shen YF Epidemiologic and clinical characteristics of 91 hospitalized patients with COVID-19 in Zhejiang, China: a retrospective, multi-centre case series. *QJM.* (2020) 113:474–81. 10.1093/qjmed/hcaa089 32181807 PMC7184349

[15] Nikčević AV Spada MM. The COVID-19 anxiety syndrome scale: development and psychometric properties. *Psychiatry Res.* (2020) 292:113322. 10.1016/j.psychres.2020.113322 32736267 PMC7375349

[16] McCrae RR Costa PT. *Personality in Adulthood: A Five-Factor Theory Perspective.* New York, NY: Guilford Press (2003).

[17] Garbe L Rau R Toppe T. Influence of perceived threat of Covid-19 and HEXACO personality traits on toilet paper stockpiling. *Plos one* (2020) 15:e0234232. 10.1371/journal.pone.0234232 32530911 PMC7292383

[18] Lee SA Crunk EA. Fear and psychopathology during the COVID-19 crisis: neuroticism, hypochondriasis, reassurance-seeking, and coronaphobia as fear factors. *OMEGA.* (2020):1–14. 10.1177/0030222820949350 32762291

[19] Somma A Gialdi G Krueger RF Markon KE Frau C Lovallo S Dysfunctional personality features, non-scientifically supported causal beliefs, and emotional problems during the first month of the COVID-19 pandemic in Italy. *Pers Ind Dif.* (2020) 165:110139. 10.1016/j.paid.2020.110139 32501318 PMC7247995

[20] Liu S Lithopoulos A Zhang CQ Garcia-Barrera MA Rhodes RE. Personality and perceived stress during COVID-19 pandemic: testing the mediating role of perceived threat and efficacy. *Pers Ind Dif.* (2021) 168:110351. 10.1016/j.paid.2020.110351 32863508 PMC7442020

[21] Shapiro SL Carlson LE Astin JA Freedman B. Mechanisms of mindfulness. *J Clin Psychology.* (2006) 62:373–86. 10.1002/jclp.20237 16385481

[22] Guendelman S Medeiros S Rampes H. Mindfulness and emotion regulation: insights from neurobiological, psychological, and clinical studies. *Front Psychol.* (2017) 8:220. 10.3389/fpsyg.2017.00220 28321194 PMC5337506

[23] Conversano C Di Giuseppe M Miccoli M Ciacchini R Gemignani A Orrù G. Mindfulness, age and gender as protective factors against psychological distress during Covid-19 pandemic. *Front Psychol.* (2020) 11:1900. 10.3389/fpsyg.2020.01900 33013503 PMC7516078

[24] O’Brien WH Horan KA Singh SR Moeller MM Wasson RS Jex SM Relationships among training, mindfulness, and workplace injuries among nurse aides working in long-term care settings. *Occup Health Sci.* (2019) 3:45–58. 10.1186/s12913-016-1423-5 27409075 PMC4943498

[25] Lyvers M Makin C Toms E Thorberg FA Samios C. Trait mindfulness in relation to emotional self-regulation and executive function. *Mindfulness.* (2014) 5:619–25.

[26] Cash M Whittingham K. What facets of mindfulness contribute to psychological well-being and depressive, anxious, and stress-related symptomatology? *Mindfulness.* (2010) 1:177–82.

[27] Derogatis LR Unger R. Symptom checklist-90-revised. In: American Cancer Society editor. *The Corsini Encyclopedia of Psychology.* New York, NY: Wiley (2010). p. 1–2. 10.1002/9780470479216.corpsy0970

[28] Di Corrado D Magnano P Muzii B Coco M Guarnera M De Lucia S Effects of social distancing on psychological state and physical activity routines during the COVID-19 pandemic. *Sport Sci Health.* (2020) 16:619–24. 10.1007/s11332-020-00697-5 32994822 PMC7517049

[29] Duncan GE Avery AR Seto E Tsang S. Perceived change in physical activity levels and mental health during COVID-19: findings among adult twin pairs. *PLoS One.* (2020) 15:e0237695. 10.1371/journal.pone.0237695 32790745 PMC7425865

[30] Cheval B Miller MW Orsholits D Berry T Sander D Boisgontier MP. Physically active individuals look for more: an eye-tracking study of attentional bias. *Psychophysiology.* (2020) 57:e13582. 10.1111/psyp.13582 32277857

[31] Ginoux C Isoard-Gautheur S Teran-Escobar C Forestier C Chalabaev A Clavel A Being active during the lockdown: the recovery potential of physical activity for well-being. *Int J Environ Res Public Health.* (2021) 18:1707. 10.3390/ijerph18041707 33578869 PMC7916567

[32] Wright LJ Williams SE Veldhuijzen van Zanten JJ. Physical activity protects against the negative impact of coronavirus fear on adolescent mental health and well-being during the COVID-19 pandemic. *Front Psychol.* (2021) 12:737. 10.3389/fpsyg.2021.580511 33776827 PMC7990778

[33] Javelle F Laborde S Hosang T Metcalfe AJ Zimmer P. The importance of nature exposure and physical activity for psychological health and stress perception: evidence from the lockdown period during the COVID-19 pandemic 2020 in France and Germany. *Front Psychol.* (2021) 12:425. 10.3389/fpsyg.2021.623946 33746842 PMC7969516

[34] Christian HE Westgarth C Bauman A Richards EA Rhodes RE Evenson KR Dog ownership and physical activity: a review of the evidence. *J Phys Act Health.* (2013) 10:750–9. 10.1123/jpah.10.5.750 23006510

[35] Levine GN Allen K Braun LT Christian HE Friedmann E Taubert KA Pet ownership and cardiovascular risk: a scientific statement from the American Heart Association. *Circulation.* (2013) 127:2353–63. 10.1161/CIR.0b013e31829201e1 23661721

[36] Morgan L Protopopova A Birkler RID Itin-Shwartz B Sutton GA Gamliel A Human–dog relationships during the COVID-19 pandemic: booming dog adoption during social isolation. *Humanit Soc Sci Commun.* (2020) 7:1–11.

[37] Bussolari C Currin-McCulloch J Packman W Kogan L Erdman P. “I couldn’t have asked for a better quarantine partner!”: experiences with companion dogs during Covid-19. *Animals.* (2021) 11:330. 10.3390/ani11020330 33525673 PMC7911354

[38] Owczarczak-Garstecka SC Graham TM Archer DC Westgarth C. Dog Walking before and during the COVID-19 pandemic lockdown: experiences of Uk dog owners. *Int J Environ Res Public Health.* (2021) 18:6315. 10.3390/ijerph18126315 34200926 PMC8296116

[39] Oliva JL Johnston KL. Puppy love in the time of Corona: dog ownership protects against loneliness for those living alone during the COVID-19 lockdown. *Int J Soc Psychiatry.* (2021) 67:232–42. 10.1177/0020764020944195 32701015 PMC7383093

[40] Deatherage S Servaty-Seib HL Aksoz I. Stress, coping, and internet use of college students. *J Am Coll Health.* (2014) 62:40–6. 24313695 10.1080/07448481.2013.843536

[41] Islam AN Laato S Talukder S Sutinen E. Misinformation sharing and social media fatigue during COVID-19: an affordance and cognitive load perspective. *Technol Forecast Soc Change.* (2020) 159:120201. 10.1016/j.techfore.2020.120201 32834137 PMC7354273

[42] Kopilaš V Hasratian AM Martinelli L Ivkić G Brajković L Gajović S. Self-perceived mental health status, digital activity and physical distancing in the context of lockdown versus not-in-lockdown measures in Italy and Croatia: cross-sectional study in early ascending phase of COVID-19 pandemic in March 2020. *Front Psychol.* (2021) 12:90. 10.3389/fpsyg.2021.621633 33613398 PMC7890192

[43] Sommerlad E David Y. Digital inequalities in times of the COVID-19 pandemic in Israel and Germany. *COVID-19 and an Emerging World of Ad Hoc Geographies*. Cham: Springer (2021).

[44] Cecchini JA Carriedo A Fernández-Río J Méndez-Giménez A González C Sánchez-Martínez B A longitudinal study on depressive symptoms and physical activity during the Spanish lockdown. *Int J Clin Health Psychol.* (2021) 21:100200. 10.1016/j.ijchp.2020.09.001 33363583 PMC7753030

[45] Ripoll J Contreras-Martos S Esteva M Soler A Serrano-Ripoll MJ. Mental health and psychological wellbeing during the COVID-19 lockdown: a longitudinal study in the Balearic islands (Spain). *J Clin Med.* (2021) 10:3191. 10.3390/jcm10143191 34300356 PMC8304509

[46] Ruggieri S Ingoglia S Bonfanti RC Coco GL. The role of online social comparison as a protective factor for psychological wellbeing: a longitudinal study during the COVID-19 quarantine. *Pers Ind Dif.* (2021) 171:110486. 10.1016/j.paid.2020.110486 33169042 PMC7610095

[47] Somma A Krueger RF Markon KE Gialdi G Colanino M Ferlito D A longitudinal study on clinically relevant self-reported depression, anxiety and acute stress features among Italian community-dwelling adults during the COVID-19 related lockdown: evidence of a predictive role for baseline dysfunctional personality dimensions. *J Affect Disord.* (2021) 282:364–71. 33421864 10.1016/j.jad.2020.12.165 PMC7834264

[48] Williams JMG Mathews A MacLeod C. The emotional Stroop task and psychopathology. *Psychol Bull.* (1996) 120:3. 8711015 10.1037/0033-2909.120.1.3

[49] Dresler T Ehlis AC Attar CH Ernst LH Tupak SV Hahn T Reliability of the emotional Stroop task: an investigation of patients with panic disorder. *J Psychiatr Res.* (2012) 46:1243–8. 10.1016/j.jpsychires.2012.06.006 22770507

[50] Ashley V Honzel N Larsen J Justus T Swick D. Attentional bias for trauma-related words: exaggerated emotional Stroop effect in Afghanistan and Iraq war veterans with PTSD. *BMC psychiatry.* (2013) 13:86. 10.1186/1471-244X-13-86 23496805 PMC3608167

[51] Mogg K Bradley BP. Attentional bias in generalized anxiety disorder versus depressive disorder. *Cogn Ther Res.* (2005) 29:29–45.

[52] Andersson G Westöö J Johansson L Carlbring P. Cognitive bias *via* the internet: a comparison of web-based and standard emotional stroop tasks in social phobia. *Cogn Behav Ther.* (2006) 35:55–62. 10.1080/16506070500372469 16500777

[53] Karademas EC Christopoulou S Dimostheni A Pavlu F. Health anxiety and cognitive interference: evidence from the application of a modified Stroop task in two studies. *Pers Ind Dif.* (2008) 44:1138–50.

[54] Fabio RA Suriano R. The influence of media exposure on anxiety and working memory during lockdown period in Italy. *Int J Environ Res Public Health.* (2021) 18:9279. 10.3390/ijerph18179279 34501866 PMC8430792

[55] Fellman D Ritakallio L Waris O Jylkkä J Laine M. Beginning of the pandemic: COVID-19-elicited anxiety as a predictor of working memory performance. *Front psychol.* (2020) 11:576466. 10.3389/fpsyg.2020.576466 33324288 PMC7725684

[56] Dalmaso M Castelli L Galfano G. Increased gaze cueing of attention during COVID-19 lockdown. *Iscience.* (2021) 24:103283. 10.1016/j.isci.2021.103283 34667942 PMC8516435

[57] Rosenholtz R. General-purpose localization of textured image regions. In: Rogowitz BE Pappas TN editors. *Proceedings of the Human Vision and Electronic Imaging IV.* (Vol. 3644), Bellingham, WA: International Society for Optics and Photonics (1999). p. 454–60. 10.1117/12.348465

[58] Rosenholtz R. Significantly different textures: a computational model of pre-attentive texture segmentation. In: Vernon D editor. *Proceedings of the European Conference on Computer Vision.* Berlin: Springer (2000). p. 197–211.

[59] Rosenholtz R. Visual search for orientation among heterogeneous distractors: experimental results and implications for signal-detection theory models of search. *J Exp Psychology.* (2001) 27:985. 10.1037//0096-1523.27.4.985 11518158

[60] Goldberg LR. The structure of phenotypic personality traits. *Am Psychol.* (1993) 48:26. 8427480 10.1037//0003-066x.48.1.26

[61] Fossati A Borroni S Marchione D Maffei C. The big five inventory (BFI). Reliability and validity of its italian translation in three independent nonclinical samples. *Eur J Psychol Assess.* (2011) 27:50–8. 10.1027/1015-5759/a000043

[62] Lovibond PF Lovibond SH. The structure of negative emotional states: comparison of the depression anxiety stress scales (DASS) with the beck depression and anxiety inventories. *Behav Res Ther.* (1995) 33:335–43. 10.1016/0005-7967(94)00075-u 7726811

[63] Antony MM Bieling PJ Cox BJ Enns MW Swinson RP. Psychometric properties of the 42-item and 21-item versions of the depression anxiety stress scales in clinical groups and a community sample. *Psychol Assess.* (1998) 10:176.

[64] Henry JD Crawford JR. The short-form version of the Depression Anxiety Stress Scales (DASS-21): construct validity and normative data in a large non-clinical sample. *Br J Clin Psychol.* (2005) 44:227–39. 10.1348/014466505X29657 16004657

[65] Bastien CH Vallières A Morin CM. Validation of the insomnia severity index as an outcome measure for insomnia research. *Sleep Med.* (2001) 2:297–307. 10.1016/s1389-9457(00)00065-4 11438246

[66] Hedman E Ljótsson B Blom K El Alaoui S Kraepelien M Rück C Telephone versus internet administration of self-report measures of social anxiety, depressive symptoms, and insomnia: psychometric evaluation of a method to reduce the impact of missing data. *J Med Internet Res.* (2013) 15:e229. 10.2196/jmir.2818 24140566 PMC3806436

[67] Morin CM Belleville G Bélanger L Ivers H. The Insomnia Severity Index: psychometric indicators to detect insomnia cases and evaluate treatment response. *Sleep.* (2011) 34:601–8. 10.1093/sleep/34.5.601 21532953 PMC3079939

[68] Weiss DS. The impact of event scale: revised. In: Wilson JP Tang CS editors. *Cross-Cultural Assessment of Psychological Trauma and PTSD.* Boston, MA: Springer (2007). p. 219–38.

[69] Creamer M Bell R Failla S. Psychometric properties of the impact of event scale—revised. *Behav Res Ther.* (2003) 41:1489–96. 14705607 10.1016/j.brat.2003.07.010

[70] Baer RA Carmody J Hunsinger M. Weekly change in mindfulness and perceived stress in a mindfulness-based stress reduction program. *J Clin Psychol.* (2012) 68:755–65. 10.1002/jclp.21865 22623334

[71] Bohlmeijer E Ten Klooster PM Fledderus M Veehof M Baer R. Psychometric properties of the five facet mindfulness questionnaire in depressed adults and development of a short form. *Assessment.* (2011) 18:308–20. 10.1177/1073191111408231 21586480

[72] Giovannini C Giromini L Bonalume L Tagini A Lang M Amadei G. The Italian five facet mindfulness questionnaire: a contribution to its validity and reliability. *J Psychopathol Behav Assess.* (2014) 36:415–23.

[73] Hardt J. The symptom checklist-27-plus (SCL-27-plus): a modern conceptualization of a traditional screening instrument. *GMS Psycho Soc Med.* (2008) 5:Doc08. PMC2736518 19742283

[74] Wittekind CE Muhtz C Moritz S Jelinek L. Performance in a blocked versus randomized emotional Stroop task in an aged, early traumatized group with and without posttraumatic stress symptoms. *J Behav Ther Exp Psychiatry.* (2017) 54:35–43. 10.1016/j.jbtep.2016.06.003 27308725

[75] Treisman AM Gelade G. A feature-integration theory of attention. *Cogn Psychol.* (1980) 12:97–136. 7351125 10.1016/0010-0285(80)90005-5

[76] Wolfe JM. What can 1 million trials tell us about visual search? *Psychol Sci.* (1998) 9:33–9. 10.1037/xhp0000012 25485661

[77] Lieb K Merklin G Rieth C Schüttler R Hess R. Preattentive information processing in schizophrenia. *Schizophrenia Res.* (1994) 14:47–56. 10.1016/0920-9964(94)90008-6 7893621

[78] Elahipanah A Christensen BK Reingold EM. Visual search performance among persons with schizophrenia as a function of target eccentricity. *Neuropsychology.* (2010) 24:192. 10.1037/a0017523 20230113

[79] Miller EK Cohen JD. An integrative theory of prefrontal cortex function. *Ann Rev Neurosci.* (2001) 24:167–202. 11283309 10.1146/annurev.neuro.24.1.167

[80] Christianson S Marren J. The impact of event scale–revised (IES-R). *Medsurg Nurs.* (2012) 21:321–3. 23243796

[81] Latkin CA Edwards C Davey-Rothwell MA Tobin KE. The relationship between social desirability bias and self-reports of health, substance use, and social network factors among urban substance users in Baltimore, Maryland. *Addict Behav.* (2017) 73:133–6. 10.1016/j.addbeh.2017.05.005 28511097 PMC5519338

[82] Wagenmakers EJ Marsman M Jamil T Ly A Verhagen J Love J Bayesian inference for psychology. Part I: theoretical advantages and practical ramifications. *Psychon Bull Rev.* (2018) 25:35–57. 10.3758/s13423-017-1343-3 28779455 PMC5862936

[83] RStudio Inc. *Integrated Development Environment for R. Version: 1.0.44.* Boston, MA: RStudio (2016).

[84] Rosseel Y. Lavaan: an R package for structural equation modeling and more. Version 0.5–12 (BETA). *J Stat Soft.* (2012) 48:1–36.

[85] Revelle WR. *Psych: Procedures for Personality and Psychological Research (Version 2.0. 9).* (2020).

[86] Wickham H Francois R Henry L Müller K. *Dplyr.* (2021).

[87] Kassambara A. *Practical Guide to Principal Component Methods in R: PCA, M (CA), FAMD, MFA, HCPC, Factoextra.* (Vol. 2). STHDA (2017).

[88] Biondi S Casale S Burrai J Mazza C Cavaggioni G Ferracuti S Personality and lockdown: a study on Italian undergraduates during the COVID-19 pandemic. *Front Psychiatry.* (2021) 12:818. 10.3389/fpsyt.2021.622366 34122161 PMC8192707

[89] Cao C Wang L Fang R Liu P Bi Y Luo S Anxiety, depression, and PTSD symptoms among high school students in china in response to the COVID-19 pandemic and lockdown. *J Affect Disord.* (2022) 296:126–9. 10.1016/j.jad.2021.09.052 34601299 PMC8456154

[90] Ozamiz-Etxebarria N Idoiaga Mondragon N Dosil Santamaría M Picaza Gorrotxategi M. Psychological symptoms during the two stages of lockdown in response to the COVID-19 outbreak: an investigation in a sample of citizens in Northern Spain. *Front psychol.* (2020) 11:1491. 32625157 10.3389/fpsyg.2020.01491 PMC7314923

[91] Stanton R To QG Khalesi S Williams SL Alley SJ Thwaite TL Depression, anxiety and stress during COVID-19: associations with changes in physical activity, sleep, tobacco and alcohol use in Australian adults. *Int J Environ Res Public Health.* (2020) 17:4065. 10.3390/ijerph17114065 32517294 PMC7312903

[92] Knolle F Ronan L Murray GK. The impact of the COVID-19 pandemic on mental health in the general population: a comparison between Germany and the UK. *BMC Psychol.* (2021) 9:60. 10.1186/s40359-021-00565-y 33892807 PMC8064888

[93] Luchetti M Lee JH Aschwanden D Sesker A Strickhouser JE Terracciano A The trajectory of loneliness in response to COVID-19. *Am Psychol.* (2020) 75:897–908. 10.1037/amp0000690 32567879 PMC7890217

[94] Medvedev ON Norden PA Krägeloh CU Siegert RJ. Investigating unique contributions of dispositional mindfulness facets to depression, anxiety, and stress in general and student populations. *Mindfulness.* (2018) 9:1757–67.

[95] Linardon J. Can acceptance, mindfulness, and self-compassion be learned by smartphone apps? A systematic and meta-analytic review of randomized controlled trials. *Behav Ther.* (2020) 51:646–58. 10.1016/j.beth.2019.10.002 32586436

[96] Lim J Leow Z Ong JC Pang LS Lim E. *The Effects of Online Group Mindfulness Training on Stress and Sleep Quality During the COVID-19 Pandemic in Singapore: a Retrospective Equivalence Trial.* (2020). Available onlibe at: https://ssrn.com/abstract=3629960 (accessed June 18, 2020). 10.2196/21757 PMC7962857 33482627

[97] Wei S Zhu F Chen X. Do stressors stifle or facilitate employees’ innovative use of enterprise systems: the moderating role of IT mindfulness. *Inform Technol People.* (2020) 34:955–77. 10.1108/ITP-09-2019-0499

[98] Király O Potenza MN Stein DJ King DL Hodgins DC Saunders JB Preventing problematic internet use during the COVID-19 pandemic: consensus guidance. *Compr Psychiatry.* (2020) 100:152180. 10.1016/j.comppsych.2020.152180 32422427 PMC7215166

[99] Moawad P Andres L. Tackling COVID-19 in informal tented settlements (Lebanon): an assessment of preparedness and response plans and their impact on the health vulnerabilities of Syrian refugees. *J Migr Health.* (2020) 1:100011. 10.1016/j.jmh.2020.100011 34405166 PMC8351976

[100] Huang Q Chen X Huang S Shao T Liao Z Lin S Substance and internet use during the COVID-19 pandemic in China. *Transl Psychiatry.* (2021) 11:1–8. 34556627 10.1038/s41398-021-01614-1 PMC8459580

[101] Young J Pritchard R Nottle C Banwell H. Pets, touch, and COVID-19: health benefits from non-human touch through times of stress. *J Behav Econ Policy.* (2020) 4:25–33.

[102] Bowen J García E Darder P Argüelles J Fatjó J. The effects of the Spanish COVID-19 lockdown on people, their pets, and the human-animal bond. *J Vet Behav.* (2020) 40:75–91. 10.1016/j.jveb.2020.05.013 32837452 PMC7292953

[103] Ratschen E Shoesmith E Shahab L Silva K Kale D Toner P Human-animal relationships and interactions during the Covid-19 lockdown phase in the UK: investigating links with mental health and loneliness. *PloS One.* (2020) 15:e0239397. 10.1371/journal.pone.0239397 32976500 PMC7518616

[104] Moore SA Faulkner G Rhodes RE Brussoni M Chulak-Bozzer T Ferguson LJ Impact of the COVID-19 virus outbreak on movement and play behaviours of Canadian children and youth: a national survey. *Int J Behav Nutr Phys Act.* (2020) 17:1–11. 10.1186/s12966-020-00987-8 32631350 PMC7336091

[105] Pouso S Borja Á Fleming LE Gómez-Baggethun E White MP Uyarra MC. Contact with blue-green spaces during the COVID-19 pandemic lockdown beneficial for mental health. *Sci Total Environ.* (2021) 756:143984. 10.1016/j.scitotenv.2020.143984 33277006 PMC7688424

[106] de Bell S White M Griffiths A Darlow A Taylor T Wheeler B Spending time in the garden is positively associated with health and wellbeing: results from a national survey in England. *Landsc Urban Plan.* (2020) 200:103836.

[107] Kingsley JY Townsend M Henderson-Wilson C. Cultivating health and wellbeing: members’ perceptions of the health benefits of a Port Melbourne community garden. *Leis Stud.* (2009) 28:207–19.

[108] Gazzetta Ufficiale. *Government of Italy Decree of the president of the Council of Ministers 10 April 2020.* (2020). Available online at: https://www.gazzettaufficiale.it/eli/id/2020/04/11/20A02179/sg (accessed May 30, 2020).

[109] Gazzetta Ufficiale. *Government of Italy Decree of the president of the Council of Ministers 9 March 2020.* (2020). Available online at: https://www.gazzettaufficiale.it/eli/id/2020/03/09/20A01558/sg (accessed May 30, 2020).

