# Supplementary material for: The Evolution of Psychological and Behavioral Consequences of Self-Isolation During Lockdown: A Longitudinal Study Across United Kingdom and Italy
Source: Front Psychiatry. 2022 Jun 1;13:826277. doi: 10.3389/fpsyt.2022.826277 (PMC9198491; doi:10.3389/fpsyt.2022.826277)
Supplement: Supplementary file 1 [file Table_2.DOCX]

# SUPPLEMENTARY MATERIALS

*Acronyms for all tables:* DASS = Depression Anxiety Stress Scale – 21 items version; ISI = Insomnia Severity Index; SCL-27 = Symptom Checklist – 27 items version; BF = Big Five Questionnaire; FFMQ = Five Factors Mindfulness Questionnaire

## Habits questionnaire

**Let’s talk about your daily routine during LAST WEEK. Please specify how much time you spent doing the following activities:**

|  | **0**  **I do not carry out this activity** | **1**  **Once a week or less** | **2**  **Few times a week** | **3**  **Less than one hour a day** | **4**  **One to three hours a day** | **5**  **More than three hours a day** |
| --- | --- | --- | --- | --- | --- | --- |
| Web browsing |  |  |  |  |  |  |
| Playing cards or board games |  |  |  |  |  |  |
| Watching movies or TV series |  |  |  |  |  |  |
| Watching YouTube videos |  |  |  |  |  |  |
| Doing high-intensity workouts (running, weights, rope) |  |  |  |  |  |  |
| Practicing yoga/pilates |  |  |  |  |  |  |
| Practicing mindfulness (or other forms of meditation) |  |  |  |  |  |  |
| Watching television |  |  |  |  |  |  |
| Reading news |  |  |  |  |  |  |
| Listening to podcast |  |  |  |  |  |  |
| Reading a book |  |  |  |  |  |  |
| Listening to the radio |  |  |  |  |  |  |
| Social networking (Facebook, Instagram, etc) |  |  |  |  |  |  |
| Calling friends/family |  |  |  |  |  |  |
| Painting / drawing / colouring |  |  |  |  |  |  |
| Listening to music |  |  |  |  |  |  |
| Playing a musical instrument |  |  |  |  |  |  |
| Learning through online courses |  |  |  |  |  |  |
| Cooking |  |  |  |  |  |  |
| Listening to audiobooks |  |  |  |  |  |  |
| Playing videogames |  |  |  |  |  |  |
| Walking the dog |  |  |  |  |  |  |

## “Going outside” questionnaire

**How many times in a week do you leave home for the following reasons?**

|  | **0**  **I do not carry out this activity** | **1**  **Once a week or less** | **2**  **Few times a week** | **3**  **Less than one hour a day** | **4**  **One to three hours a day** | **5**  **More than three hours a day** |
| --- | --- | --- | --- | --- | --- | --- |
| Buying essential groceries |  |  |  |  |  |  |
| Buying medicines |  |  |  |  |  |  |
| Going to work |  |  |  |  |  |  |
| Walking the dog |  |  |  |  |  |  |
| Buying the newspaper |  |  |  |  |  |  |
| Buying cigars or cigarettes |  |  |  |  |  |  |
| Exercising outside (i.e. walking, jogging) |  |  |  |  |  |  |

## Model parameters

Parameters in bold are statistically significant according to the α = 0.0125 threshold.

| T1 | | | | |
| --- | --- | --- | --- | --- |
| **REGRESSIONS** | | | | |
|  | **Estimate** | **Std. Err** | **z value** | **P (>\|z\|)** |
| DASS: Depression ~ | | | | |
| BF: Extraversion | -0.208 | 0.160 | -1.297 | 0.194 |
| BF: Agreeableness | -0.128 | 0.187 | -0.686 | 0.493 |
| BF: Conscientiousness | -0.341 | 0.162 | -2.107 | 0.035 |
| **BF: Neuroticism** | **0.706** | **0.222** | **3.182** | **0.001** |
| **BF: Openness** | **-0.891** | **0.209** | **-4.263** | **< 0.001** |
| FFMQ: Observation | 1.025 | 0.523 | 1.959 | 0.050 |
| **FFMQ: Description** | **1.082** | **0.407** | **2.656** | **0.008** |
| FFMQ: Aware actions | 0.196 | 0.494 | 0.396 | 0.692 |
| FFMQ: Non-judgment | -0.798 | 0.417 | -1.913 | 0.056 |
| FFMQ: Nonreactivity | -0.737 | 0.409 | -1.802 | 0.072 |
| Household | -0.674 | 0.667 | -1.012 | 0.312 |
| House size | 0.085 | 1.017 | 0.083 | 0.934 |
| Garden | -2.646 | 1.955 | -1.353 | 0.176 |
| Routine: Component 1 | -1.848 | 1.080 | -1.710 | 0.087 |
| Routine: Component 2 | -1.025 | 0.956 | -1.071 | 0.284 |
| Routine: Component 3 | -0.688 | 0.836 | -0.823 | 0.410 |
| Routine: Component 4 | 2.346 | 1.088 | 2.157 | 0.031 |
| Routine: Component 5 | -0.139 | 1.092 | -0.127 | 0.899 |
| Going outside: Component 1 | -0.498 | 1.395 | -0.357 | 0.721 |
| Going outside: Component 2 | -1.299 | 0.829 | -1.567 | 0.117 |
| Going outside: Component 3 | -0.113 | 0.919 | -0.123 | 0.902 |
| Going outside: Component 4 | 2.011 | 0.891 | 2.258 | 0.024 |
| Balcony | -1.087 | 1.911 | -0.569 | 0.569 |
| Nicotine | -0.039 | 0.050 | -0.766 | 0.444 |
| Days in lockdown | 0.112 | 0.093 | 1.197 | 0.231 |
|  | | | | |
|  | **Estimate** | **Std. Err** | **z value** | **P (>\|z\|)** |
| DASS: Anxiety ~ | | | | |
| BF: Extraversion | 0.118 | 0.136 | 0.863 | 0.388 |
| BF: Agreeableness | 0.015 | 0.159 | 0.093 | 0.926 |
| BF: Conscientiousness | -0.223 | 0.138 | -1.618 | 0.106 |
| **BF: Neuroticism** | **0.518** | **0.189** | **2.747** | **0.006** |
| **BF: Openness** | **-0.488** | **0.178** | **-2.749** | **0.006** |
| FFMQ: Observation | 0.565 | 0.445 | 1.271 | 0.204 |
| FFMQ: Description | 0.388 | 0.346 | 1.119 | 0.263 |
| FFMQ: Aware actions | -0.516 | 0.420 | -1.228 | 0.220 |
| FFMQ: Non-judgment | 0.218 | 0.355 | 0.616 | 0.538 |
| FFMQ: Nonreactivity | -0.280 | 0.348 | -0.806 | 0.420 |
| Household | 0.999 | 0.567 | 1.762 | 0.078 |
| House size | 0.470 | 0.865 | 0.543 | 0.587 |
| Garden | -1.342 | 1.662 | -0.808 | 0.419 |
| Routine: Component 1 | -0.097 | 0.918 | -0.106 | 0.916 |
| Routine: Component 2 | 1.364 | 0.813 | 1.678 | 0.093 |
| Routine: Component 3 | 0.634 | 0.711 | 0.892 | 0.372 |
| Routine: Component 4 | 0.363 | 0.925 | 0.393 | 0.694 |
| Routine: Component 5 | -0.104 | 0.929 | -0.112 | 0.911 |
| Going outside: Component 1 | -0.843 | 1.186 | -0.711 | 0.477 |
| Going outside: Component 2 | -0.435 | 0.704 | -0.618 | 0.537 |
| Going outside: Component 3 | -0.046 | 0.782 | -0.059 | 0.953 |
| Going outside: Component 4 | 0.190 | 0.757 | 0.250 | 0.802 |
| Balcony | -2.925 | 1.624 | -1.801 | 0.072 |
| Nicotine | 0.038 | 0.043 | 0.886 | 0.376 |
| Days in lockdown | 0.007 | 0.079 | 0.094 | 0.925 |
|  | | | | |
|  | **Estimate** | **Std. Err** | **z value** | **P (>\|z\|)** |
| DASS: Stress ~ | | | | |
| BF: Extraversion | -0.080 | 0.175 | -0.458 | 0.647 |
| BF: Agreeableness | 0.171 | 0.204 | 0.840 | 0.401 |
| **BF: Conscientiousness** | **-0.484** | **0.177** | **-2.737** | **0.006** |
| **BF: Neuroticism** | **0.959** | **0.242** | **3.965** | **< 0.001** |
| **BF: Openness** | **-0.858** | **0.228** | **-3.765** | **< 0.001** |
| FFMQ: Observation | 0.179 | 0.571 | 0.313 | 0.754 |
| FFMQ: Description | 0.796 | 0.444 | 1.790 | 0.073 |
| FFMQ: Aware actions | -0.134 | 0.539 | -0.249 | 0.803 |
| FFMQ: Non-judgment | -0.843 | 0.455 | -1.853 | 0.064 |
| FFMQ: Nonreactivity | -0.661 | 0.446 | -1.482 | 0.138 |
| Household | 0.283 | 0.727 | 0.390 | 0.697 |
| House size | -0.841 | 1.110 | -0.758 | 0.448 |
| Garden | -4.218 | 2.132 | -1.978 | 0.048 |
| Routine: Component 1 | -1.108 | 1.179 | -0.940 | 0.347 |
| Routine: Component 2 | -0.834 | 1.043 | -0.800 | 0.424 |
| Routine: Component 3 | 1.466 | 0.912 | 1.608 | 0.108 |
| Routine: Component 4 | 0.247 | 1.187 | 0.208 | 0.835 |
| Routine: Component 5 | -0.325 | 1.191 | -0.273 | 0.785 |
| Going outside: Component 1 | 0.844 | 1.522 | 0.555 | 0.579 |
| Going outside: Component 2 | 0.322 | 0.904 | 0.357 | 0.721 |
| Going outside: Component 3 | 1.838 | 1.003 | 1.833 | 0.067 |
| Going outside: Component 4 | 1.568 | 0.972 | 1.613 | 0.107 |
| Balcony | -0.606 | 2.084 | -0.291 | 0.771 |
| Nicotine | 0.030 | 0.055 | 0.537 | 0.591 |
| Days in lockdown | -0.093 | 0.102 | -0.912 | 0.362 |
|  | | | | |
|  | **Estimate** | **Std. Err** | **z value** | **P (>\|z\|)** |
| SCL-27: Total score~ | | | | |
| BF: Extraversion | -0.006 | 0.009 | -0.643 | 0.520 |
| BF: Agreeableness | -0.005 | 0.011 | -0.453 | 0.650 |
| BF: Conscientiousness | -0.019 | 0.009 | -1.983 | 0.047 |
| **BF: Neuroticism** | **0.051** | **0.012** | **4.242** | **< 0.001** |
| **BF: Openness** | **-0.039** | **0.012** | **-3.206** | **0.001** |
| FFMQ: Observation | -0.011 | 0.031 | -0.365 | 0.715 |
| FFMQ: Description | 0.057 | 0.024 | 2.376 | 0.018 |
| FFMQ: Aware actions | -0.007 | 0.027 | -0.255 | 0.798 |
| FFMQ: Non-judgment | -0.041 | 0.024 | -1.728 | 0.084 |
| FFMQ: Nonreactivity | 0.013 | 0.024 | 0.550 | 0.583 |
| Household | 0.052 | 0.036 | 1.417 | 0.156 |
| House size | 0.034 | 0.060 | 0.569 | 0.569 |
| Garden | -0.167 | 0.113 | -1.479 | 0.139 |
| Routine: Component 1 | 0.045 | 0.060 | 0.744 | 0.457 |
| Routine: Component 2 | 0.045 | 0.056 | 0.795 | 0.427 |
| Routine: Component 3 | -0.004 | 0.049 | -0.089 | 0.929 |
| Routine: Component 4 | 0.102 | 0.063 | 1.629 | 0.103 |
| Routine: Component 5 | 0.012 | 0.064 | 0.194 | 0.846 |
| Going outside: Component 1 | 0.022 | 0.073 | 0.299 | 0.765 |
| Going outside: Component 2 | -0.089 | 0.049 | -1.830 | 0.067 |
| Going outside: Component 3 | 0.016 | 0.053 | 0.292 | 0.770 |
| Going outside: Component 4 | 0.063 | 0.046 | 1.366 | 0.172 |
| Balcony | -0.067 | 0.110 | -0.608 | 0.543 |
| Nicotine | -0.002 | 0.003 | -0.728 | 0.467 |
| Days in lockdown | -0.010 | 0.005 | -1.897 | 0.058 |
|  | | | | |
| **COVARIANCES** | | | | |
|  | **Estimate** | **Std. Err** | **z value** | **P (>\|z\|)** |
| DASS: Depression ~~ | | | | |
| DASS: Anxiety | 4.040 | 3.270 | 1.236 | 0.217 |
| **DASS: Stress** | **17.317** | **4.684** | **3.697** | **< 0.001** |
| DASS: Anxiety ~~ | | | | |
| **DASS: Stress** | **9.920** | **3.738** | **2.654** | **0.008** |
|  | | | | |
| **VARIANCES** | | | | |
|  | **Estimate** | **Std. Err** | **z value** | **P (>\|z\|)** |
| **DASS: Depression** | **30.157** | **5.373** | **5.612** | **< 0.001** |
| **DASS: Anxiety** | **21.792** | **3.883** | **5.612** | **< 0.001** |
| **DASS: Stress** | **35.881** | **6.393** | **5.612** | **< 0.001** |
| **SCL-27: Total score** | **0.104** | **0.018** | **5.657** | **< 0.001** |
|  | | | | |
| **R-SQUARE** | |  |  |  |
|  | **Estimate** |  |  |  |
| **DASS: Depression** | **0.695** |  |  |  |
| **DASS: Anxiety** | **0.595** |  |  |  |
| **DASS: Stress** | **0.626** |  |  |  |
| **SCL-27: Total score** | **0.669** |  |  |  |

| T2 | | | | |
| --- | --- | --- | --- | --- |
| **REGRESSIONS** | | | | |
|  | **Estimate** | **Std. Err** | **z value** | **P (>\|z\|)** |
| DASS: Depression ~ | | | | |
| BF: Extraversion | 0.134 | 0.237 | 0.565 | 0.572 |
| BF: Agreeableness | 0.064 | 0.297 | 0.217 | 0.828 |
| BF: Conscientiousness | -0.048 | 0.209 | -0.229 | 0.819 |
| BF: Neuroticism | 0.377 | 0.306 | 1.233 | 0.218 |
| BF: Openness | -0.371 | 0.315 | -1.178 | 0.239 |
| FFMQ: Observation | 0.546 | 0.557 | 0.982 | 0.326 |
| FFMQ: Description | 0.077 | 0.617 | 0.125 | 0.901 |
| FFMQ: Aware actions | -0.699 | 0.600 | -1.166 | 0.244 |
| **FFMQ: Non-judgment** | **-1.290** | **0.515** | **-2.503** | **0.012** |
| FFMQ: Nonreactivity | -0.787 | 0.544 | -1.446 | 0.148 |
| Household | -1.300 | 0.871 | -1.494 | 0.135 |
| House size | -0.320 | 1.377 | -0.232 | 0.816 |
| Garden | -1.393 | 2.684 | -0.519 | 0.604 |
| Routine: Component 1 | -0.890 | 1.367 | -0.651 | 0.515 |
| Routine: Component 2 | -0.138 | 1.426 | -0.097 | 0.923 |
| Routine: Component 3 | -1.937 | 1.437 | -1.347 | 0.178 |
| Routine: Component 4 | 1.036 | 1.239 | 0.836 | 0.403 |
| Routine: Component 5 | -1.289 | 1.756 | -0.734 | 0.463 |
| Going outside: Component 1 | -0.545 | 1.707 | -0.320 | 0.749 |
| Going outside: Component 2 | 1.584 | 1.300 | 1.219 | 0.223 |
| Going outside: Component 3 | -0.493 | 1.240 | -0.398 | 0.691 |
| Going outside: Component 4 | 1.996 | 1.228 | 1.626 | 0.104 |
| Balcony | -0.008 | 2.692 | -0.003 | 0.998 |
| Nicotine | -0.029 | 0.052 | -0.568 | 0.570 |
| **Days in lockdown** | **0.330** | **0.097** | **3.394** | **0.001** |
|  | | | | |
|  | **Estimate** | **Std. Err** | **z value** | **P (>\|z\|)** |
| DASS: Anxiety ~ | | | | |
| BF: Extraversion | 0.165 | 0.141 | 1.168 | 0.243 |
| BF: Agreeableness | -0.174 | 0.177 | -0.982 | 0.326 |
| BF: Conscientiousness | -0.222 | 0.125 | -1.776 | 0.076 |
| BF: Neuroticism | 0.204 | 0.182 | 1.120 | 0.263 |
| BF: Openness | -0.428 | 0.188 | -2.281 | 0.023 |
| FFMQ: Observation | 0.540 | 0.332 | 1.627 | 0.104 |
| FFMQ: Description | 0.283 | 0.368 | 0.769 | 0.442 |
| FFMQ: Aware actions | -0.589 | 0.358 | -1.647 | 0.100 |
| FFMQ: Non-judgment | 0.657 | 0.307 | 2.139 | 0.032 |
| FFMQ: Nonreactivity | 0.047 | 0.324 | 0.144 | 0.886 |
| Household | 0.537 | 0.519 | 1.035 | 0.301 |
| House size | 1.129 | 0.821 | 1.376 | 0.169 |
| Garden | -1.490 | 1.600 | -0.931 | 0.352 |
| Routine: Component 1 | -0.322 | 0.815 | -0.396 | 0.692 |
| Routine: Component 2 | 1.029 | 0.850 | 1.211 | 0.226 |
| Routine: Component 3 | -0.075 | 0.857 | -0.087 | 0.930 |
| Routine: Component 4 | -1.314 | 0.739 | -1.780 | 0.075 |
| Routine: Component 5 | 0.262 | 1.047 | 0.250 | 0.803 |
| Going outside: Component 1 | -1.415 | 1.018 | -1.390 | 0.164 |
| Going outside: Component 2 | 1.411 | 0.775 | 1.821 | 0.069 |
| Going outside: Component 3 | -0.810 | 0.739 | -1.096 | 0.273 |
| Going outside: Component 4 | -1.251 | 0.732 | -1.709 | 0.087 |
| **Balcony** | **-4.345** | **1.605** | **-2.708** | **0.007** |
| Nicotine | 0.033 | 0.031 | 1.058 | 0.290 |
| Days in lockdown | 0.054 | 0.058 | 0.927 | 0.354 |
|  | | | | |
|  | **Estimate** | **Std. Err** | **z value** | **P (>\|z\|)** |
| DASS: Stress ~ | | | | |
| BF: Extraversion | -0.016 | 0.249 | -0.063 | 0.950 |
| BF: Agreeableness | 0.075 | 0.312 | 0.239 | 0.811 |
| BF: Conscientiousness | -0.120 | 0.220 | -0.545 | 0.586 |
| BF: Neuroticism | 0.557 | 0.321 | 1.734 | 0.083 |
| BF: Openness | -0.411 | 0.330 | -1.245 | 0.213 |
| FFMQ: Observation | -0.015 | 0.584 | -0.026 | 0.979 |
| FFMQ: Description | 0.483 | 0.647 | 0.747 | 0.455 |
| FFMQ: Aware actions | -0.762 | 0.629 | -1.211 | 0.226 |
| FFMQ: Non-judgment | -0.208 | 0.541 | -0.385 | 0.701 |
| FFMQ: Nonreactivity | -0.825 | 0.571 | -1.445 | 0.148 |
| Household | 0.430 | 0.913 | 0.471 | 0.638 |
| House size | -0.596 | 1.444 | -0.412 | 0.680 |
| Garden | -1.322 | 2.816 | -0.469 | 0.639 |
| Routine: Component 1 | -0.309 | 1.434 | -0.215 | 0.829 |
| Routine: Component 2 | -0.234 | 1.496 | -0.156 | 0.876 |
| Routine: Component 3 | 0.219 | 1.508 | 0.145 | 0.885 |
| Routine: Component 4 | 0.011 | 1.300 | 0.009 | 0.993 |
| Routine: Component 5 | -1.219 | 1.842 | -0.662 | 0.508 |
| Going outside: Component 1 | 0.322 | 1.791 | 0.180 | 0.857 |
| Going outside: Component 2 | 3.352 | 1.364 | 2.457 | 0.014 |
| Going outside: Component 3 | -0.283 | 1.301 | -0.217 | 0.828 |
| Going outside: Component 4 | 1.742 | 1.289 | 1.352 | 0.176 |
| Balcony | 1.092 | 2.824 | 0.387 | 0.699 |
| Nicotine | -0.010 | 0.054 | -0.177 | 0.859 |
| Days in lockdown | 0.164 | 0.102 | 1.603 | 0.109 |
|  | | | | |
|  | **Estimate** | **Std. Err** | **z value** | **P (>\|z\|)** |
| SCL-27: Total score~ | | | | |
| BF: Extraversion | 0.016 | 0.010 | 1.542 | 0.123 |
| BF: Agreeableness | 0.007 | 0.013 | 0.566 | 0.572 |
| BF: Conscientiousness | -0.010 | 0.009 | -1.064 | 0.287 |
| BF: Neuroticism | 0.031 | 0.013 | 2.375 | 0.018 |
| **BF: Openness** | **-0.036** | **0.014** | **-2.629** | **0.009** |
| FFMQ: Observation | 0.045 | 0.024 | 1.886 | 0.059 |
| FFMQ: Description | 0.020 | 0.027 | 0.761 | 0.447 |
| FFMQ: Aware actions | -0.043 | 0.026 | -1.674 | 0.094 |
| FFMQ: Non-judgment | -0.031 | 0.022 | -1.385 | 0.166 |
| FFMQ: Nonreactivity | -0.030 | 0.023 | -1.265 | 0.206 |
| Household | 0.046 | 0.038 | 1.230 | 0.219 |
| House size | -0.025 | 0.059 | -0.422 | 0.673 |
| Garden | -0.244 | 0.116 | -2.107 | 0.035 |
| Routine: Component 1 | -0.063 | 0.059 | -1.078 | 0.281 |
| Routine: Component 2 | -0.018 | 0.061 | -0.295 | 0.768 |
| Routine: Component 3 | -0.141 | 0.062 | -2.271 | 0.023 |
| Routine: Component 4 | -0.064 | 0.053 | -1.201 | 0.230 |
| Routine: Component 5 | 0.031 | 0.076 | 0.413 | 0.680 |
| Going outside: Component 1 | -0.111 | 0.074 | -1.514 | 0.130 |
| Going outside: Component 2 | 0.006 | 0.056 | 0.105 | 0.916 |
| Going outside: Component 3 | 0.028 | 0.053 | 0.528 | 0.597 |
| Going outside: Component 4 | -0.004 | 0.053 | -0.069 | 0.945 |
| Balcony | -0.074 | 0.116 | -0.640 | 0.522 |
| Nicotine | 0.002 | 0.002 | 0.891 | 0.373 |
| Days in lockdown | -0.002 | 0.004 | -0.399 | 0.690 |
|  | | | | |
| **COVARIANCES** | | | | |
|  | **Estimate** | **Std. Err** | **z value** | **P (>\|z\|)** |
| DASS: Depression ~~ | | | | |
| **DASS: Anxiety** | **13.757** | **3.914** | **3.514** | **< 0.001** |
| **DASS: Stress** | **30.880** | **7.414** | **4.165** | **< 0.001** |
| DASS: Anxiety ~~ | | | | |
| **DASS: Stress** | **17.761** | **4.365** | **4.069** | **< 0.001** |
|  | | | | |
| **VARIANCES** | | | | |
|  | **Estimate** | **Std. Err** | **z value** | **P (>\|z\|)** |
| **DASS: Depression** | **39.752** | **8.031** | **4.950** | **< 0.001** |
| **DASS: Anxiety** | **14.127** | **2.854** | **4.950** | **< 0.001** |
| **DASS: Stress** | **43.767** | **8.842** | **4.950** | **< 0.001** |
| **SCL-27: Total score** | **0.074** | **0.015** | **4.950** | **< 0.001** |
|  | | | | |
| **R-SQUARE** | |  |  |  |
|  | **Estimate** |  |  |  |
| **DASS: Depression** | **0.546** |  |  |  |
| **DASS: Anxiety** | **0.557** |  |  |  |
| **DASS: Stress** | **0.442** |  |  |  |
| **SCL-27: Total score** | **0.696** |  |  |  |

| T3 | | | | |
| --- | --- | --- | --- | --- |
| **REGRESSIONS** | | | | |
|  | **Estimate** | **Std. Err** | **z value** | **P (>\|z\|)** |
| DASS: Depression ~ | | | | |
| BF: Extraversion | 0.315 | 0.271 | 1.162 | 0.245 |
| BF: Agreeableness | 0.233 | 0.355 | 0.657 | 0.511 |
| BF: Conscientiousness | -0.183 | 0.255 | -0.718 | 0.473 |
| BF: Neuroticism | 0.666 | 0.450 | 1.480 | 0.139 |
| BF: Openness | -0.313 | 0.366 | -0.855 | 0.393 |
| FFMQ: Observation | 0.622 | 0.686 | 0.907 | 0.364 |
| FFMQ: Description | 0.324 | 0.597 | 0.543 | 0.587 |
| FFMQ: Aware actions | 1.386 | 1.019 | 1.361 | 0.174 |
| **FFMQ: Non-judgment** | **-1.552** | **0.572** | **-2.713** | **0.007** |
| FFMQ: Nonreactivity | -0.663 | 0.525 | -1.263 | 0.206 |
| Household | -0.138 | 1.138 | -0.121 | 0.904 |
| House size | -0.389 | 1.528 | -0.255 | 0.799 |
| Garden | -0.974 | 3.132 | -0.311 | 0.756 |
| Routine: Component 1 | 0.624 | 1.425 | 0.438 | 0.661 |
| Routine: Component 2 | 0.665 | 1.245 | 0.534 | 0.593 |
| Routine: Component 3 | -2.647 | 1.535 | -1.725 | 0.085 |
| Routine: Component 4 | 1.121 | 1.534 | 0.731 | 0.465 |
| Routine: Component 5 | 0.588 | 2.117 | 0.278 | 0.781 |
| Going outside: Component 1 | -0.077 | 1.809 | -0.042 | 0.966 |
| Going outside: Component 2 | 0.385 | 1.651 | 0.233 | 0.816 |
| Going outside: Component 3 | 1.578 | 1.532 | 1.030 | 0.303 |
| Going outside: Component 4 | 2.005 | 1.289 | 1.555 | 0.120 |
| Balcony | -0.960 | 3.948 | -0.243 | 0.808 |
| Nicotine | -0.041 | 0.069 | -0.591 | 0.555 |
| Days in lockdown | 0.089 | 0.102 | 0.875 | 0.381 |
|  | | | | |
|  | **Estimate** | **Std. Err** | **z value** | **P (>\|z\|)** |
| DASS: Anxiety ~ | | | | |
| BF: Extraversion | 0.271 | 0.141 | 1.917 | 0.055 |
| BF: Agreeableness | -0.196 | 0.185 | -1.058 | 0.290 |
| BF: Conscientiousness | -0.180 | 0.133 | -1.354 | 0.176 |
| BF: Neuroticism | 0.302 | 0.235 | 1.285 | 0.199 |
| BF: Openness | -0.197 | 0.191 | -1.030 | 0.303 |
| FFMQ: Observation | 0.591 | 0.358 | 1.651 | 0.099 |
| FFMQ: Description | -0.301 | 0.312 | -0.964 | 0.335 |
| FFMQ: Aware actions | 0.170 | 0.532 | 0.320 | 0.749 |
| FFMQ: Non-judgment | 0.288 | 0.299 | 0.965 | 0.335 |
| FFMQ: Nonreactivity | -0.187 | 0.274 | -0.683 | 0.495 |
| Household | 0.063 | 0.594 | 0.107 | 0.915 |
| House size | -0.040 | 0.798 | -0.050 | 0.960 |
| Garden | 2.089 | 1.636 | 1.276 | 0.202 |
| Routine: Component 1 | 0.656 | 0.744 | 0.882 | 0.378 |
| Routine: Component 2 | 0.972 | 0.650 | 1.494 | 0.135 |
| Routine: Component 3 | -1.142 | 0.802 | -1.424 | 0.154 |
| Routine: Component 4 | -0.668 | 0.802 | -0.833 | 0.405 |
| Routine: Component 5 | 1.222 | 1.106 | 1.105 | 0.269 |
| Going outside: Component 1 | 1.784 | 0.945 | 1.887 | 0.059 |
| Going outside: Component 2 | -0.636 | 0.863 | -0.738 | 0.461 |
| Going outside: Component 3 | 0.042 | 0.801 | 0.052 | 0.958 |
| Going outside: Component 4 | 0.530 | 0.674 | 0.786 | 0.432 |
| Balcony | 0.592 | 2.063 | 0.287 | 0.774 |
| Nicotine | -0.049 | 0.036 | -1.353 | 0.176 |
| Days in lockdown | 0.025 | 0.053 | 0.465 | 0.642 |
|  | | | | |
|  | **Estimate** | **Std. Err** | **z value** | **P (>\|z\|)** |
| DASS: Stress ~ | | | | |
| BF: Extraversion | 0.067 | 0.302 | 0.222 | 0.824 |
| BF: Agreeableness | -0.023 | 0.395 | -0.058 | 0.954 |
| BF: Conscientiousness | -0.369 | 0.284 | -1.302 | 0.193 |
| BF: Neuroticism | 0.888 | 0.501 | 1.773 | 0.076 |
| BF: Openness | 0.105 | 0.408 | 0.256 | 0.798 |
| FFMQ: Observation | 0.060 | 0.763 | 0.079 | 0.937 |
| FFMQ: Description | 0.107 | 0.665 | 0.160 | 0.873 |
| FFMQ: Aware actions | 0.756 | 1.134 | 0.666 | 0.505 |
| FFMQ: Non-judgment | -0.144 | 0.637 | -0.227 | 0.821 |
| FFMQ: Nonreactivity | -0.548 | 0.585 | -0.937 | 0.349 |
| Household | 0.726 | 1.267 | 0.573 | 0.566 |
| House size | -0.112 | 1.701 | -0.066 | 0.948 |
| Garden | 0.362 | 3.487 | 0.104 | 0.917 |
| Routine: Component 1 | 0.294 | 1.586 | 0.185 | 0.853 |
| Routine: Component 2 | 2.078 | 1.386 | 1.499 | 0.134 |
| Routine: Component 3 | -1.837 | 1.709 | -1.075 | 0.282 |
| Routine: Component 4 | -0.029 | 1.708 | -0.017 | 0.987 |
| Routine: Component 5 | -1.161 | 2.357 | -0.493 | 0.622 |
| Going outside: Component 1 | 0.481 | 2.014 | 0.239 | 0.811 |
| Going outside: Component 2 | 2.388 | 1.838 | 1.299 | 0.194 |
| Going outside: Component 3 | 0.007 | 1.706 | 0.004 | 0.997 |
| Going outside: Component 4 | 2.244 | 1.436 | 1.563 | 0.118 |
| Balcony | -2.572 | 4.395 | -0.585 | 0.558 |
| Nicotine | 0.109 | 0.077 | 1.419 | 0.156 |
| Days in lockdown | 0.050 | 0.113 | 0.439 | 0.661 |
|  | | | | |
|  | **Estimate** | **Std. Err** | **z value** | **P (>\|z\|)** |
| SCL-27: Total score~ | | | | |
| BF: Extraversion | 0.006 | 0.013 | 0.500 | 0.617 |
| BF: Agreeableness | 0.002 | 0.017 | 0.127 | 0.899 |
| BF: Conscientiousness | -0.009 | 0.012 | -0.735 | 0.462 |
| BF: Neuroticism | 0.039 | 0.021 | 1.837 | 0.066 |
| BF: Openness | -0.017 | 0.017 | -0.954 | 0.340 |
| FFMQ: Observation | 0.035 | 0.033 | 1.074 | 0.283 |
| FFMQ: Description | 0.006 | 0.028 | 0.203 | 0.839 |
| FFMQ: Aware actions | 0.057 | 0.048 | 1.169 | 0.242 |
| FFMQ: Non-judgment | -0.059 | 0.027 | -2.168 | 0.030 |
| FFMQ: Nonreactivity | -0.038 | 0.025 | -1.541 | 0.123 |
| Household | 0.013 | 0.054 | 0.238 | 0.812 |
| House size | -0.044 | 0.073 | -0.608 | 0.543 |
| Garden | 0.056 | 0.149 | 0.373 | 0.709 |
| Routine: Component 1 | 0.049 | 0.068 | 0.722 | 0.470 |
| Routine: Component 2 | 0.010 | 0.059 | 0.171 | 0.864 |
| Routine: Component 3 | -0.148 | 0.073 | -2.023 | 0.043 |
| Routine: Component 4 | 0.025 | 0.073 | 0.344 | 0.731 |
| Routine: Component 5 | 0.036 | 0.101 | 0.363 | 0.717 |
| Going outside: Component 1 | 0.066 | 0.086 | 0.764 | 0.445 |
| Going outside: Component 2 | 0.056 | 0.078 | 0.708 | 0.479 |
| Going outside: Component 3 | 0.044 | 0.073 | 0.600 | 0.548 |
| Going outside: Component 4 | 0.059 | 0.061 | 0.959 | 0.337 |
| Balcony | -0.056 | 0.188 | -0.297 | 0.766 |
| Nicotine | -0.001 | 0.003 | -0.422 | 0.673 |
| Days in lockdown | 0.001 | 0.005 | 0.200 | 0.842 |
|  | | | | |
| **COVARIANCES** | | | | |
|  | **Estimate** | **Std. Err** | **z value** | **P (>\|z\|)** |
| DASS: Depression ~~ | | | | |
| **DASS: Anxiety** | **17.530** | **4.503** | **3.893** | **< 0.001** |
| **DASS: Stress** | **47.489** | **10.470** | **4.536** | **< 0.001** |
| DASS: Anxiety ~~ | | | | |
| **DASS: Stress** | **21.518** | **5.178** | **4.156** | **< 0.001** |
|  | | | | |
| **VARIANCES** | | | | |
|  | **Estimate** | **Std. Err** | **z value** | **P (>\|z\|)** |
| **DASS: Depression** | **50.139** | **10.130** | **4.950** | **< 0.001** |
| **DASS: Anxiety** | **13.686** | **2.765** | **4.950** | **< 0.001** |
| **DASS: Stress** | **62.149** | **12.556** | **4.950** | **< 0.001** |
| **SCL-27: Total score** | **0.113** | **0.023** | **4.950** | **< 0.001** |
|  | | | | |
| **R-SQUARE** | |  |  |  |
|  | **Estimate** |  |  |  |
| **DASS: Depression** | **0.472** |  |  |  |
| **DASS: Anxiety** | **0.451** |  |  |  |
| **DASS: Stress** | **0.411** |  |  |  |
| **SCL-27: Total score** | **0.529** |  |  |  |

| T4 | | | | |
| --- | --- | --- | --- | --- |
| **REGRESSIONS** | | | | |
|  | **Estimate** | **Std. Err** | **z value** | **P (>\|z\|)** |
| DASS: Depression ~ | | | | |
| BF: Extraversion | -0.617 | 0.373 | -1.652 | 0.099 |
| BF: Agreeableness | 0.115 | 0.322 | 0.356 | 0.722 |
| BF: Conscientiousness | 0.001 | 0.289 | 0.003 | 0.998 |
| BF: Neuroticism | 0.687 | 0.439 | 1.565 | 0.118 |
| BF: Openness | 0.456 | 0.382 | 1.195 | 0.232 |
| FFMQ: Observation | 0.888 | 0.778 | 1.140 | 0.254 |
| FFMQ: Description | -0.131 | 1.204 | -0.109 | 0.913 |
| FFMQ: Aware actions | 0.802 | 0.925 | 0.868 | 0.385 |
| FFMQ: Non-judgment | -1.280 | 0.641 | -1.996 | 0.046 |
| FFMQ: Nonreactivity | -1.453 | 0.786 | -1.849 | 0.065 |
| Household | 1.492 | 1.083 | 1.377 | 0.168 |
| House size | -1.936 | 1.527 | -1.267 | 0.205 |
| Garden | 0.167 | 4.120 | 0.041 | 0.968 |
| Routine: Component 1 | 0.244 | 1.807 | 0.135 | 0.893 |
| Routine: Component 2 | -1.889 | 1.746 | -1.082 | 0.279 |
| **Routine: Component 3** | **5.135** | **1.670** | **3.075** | **0.002** |
| Routine: Component 4 | 7.413 | 3.022 | 2.453 | 0.014 |
| Routine: Component 5 | -4.707 | 2.311 | -2.037 | 0.042 |
| Going outside: Component 1 | 4.558 | 2.955 | 1.542 | 0.123 |
| Going outside: Component 2 | 0.805 | 1.559 | 0.516 | 0.606 |
| Going outside: Component 3 | -3.312 | 2.036 | -1.626 | 0.104 |
| Going outside: Component 4 | -0.706 | 1.880 | -0.375 | 0.707 |
| Balcony | -0.006 | 6.235 | -0.001 | 0.999 |
| Nicotine | -0.250 | 0.145 | -1.729 | 0.084 |
| Days in lockdown | 0.166 | 0.093 | 1.785 | 0.074 |
|  | | | | |
|  | **Estimate** | **Std. Err** | **z value** | **P (>\|z\|)** |
| DASS: Anxiety ~ | | | | |
| BF: Extraversion | 0.083 | 0.225 | 0.370 | 0.711 |
| BF: Agreeableness | 0.212 | 0.194 | 1.095 | 0.274 |
| BF: Conscientiousness | -0.313 | 0.174 | -1.799 | 0.072 |
| BF: Neuroticism | 0.187 | 0.264 | 0.708 | 0.479 |
| BF: Openness | -0.032 | 0.230 | -0.140 | 0.888 |
| FFMQ: Observation | 0.334 | 0.468 | 0.713 | 0.476 |
| FFMQ: Description | -0.124 | 0.724 | -0.171 | 0.864 |
| FFMQ: Aware actions | 0.289 | 0.556 | 0.520 | 0.603 |
| FFMQ: Non-judgment | -0.080 | 0.386 | -0.208 | 0.835 |
| FFMQ: Nonreactivity | -0.274 | 0.473 | -0.579 | 0.563 |
| Household | 0.344 | 0.652 | 0.528 | 0.598 |
| House size | 0.559 | 0.919 | 0.608 | 0.543 |
| Garden | -0.378 | 2.479 | -0.153 | 0.879 |
| Routine: Component 1 | 0.342 | 1.087 | 0.314 | 0.753 |
| Routine: Component 2 | -0.150 | 1.050 | -0.142 | 0.887 |
| Routine: Component 3 | 0.330 | 1.005 | 0.329 | 0.742 |
| Routine: Component 4 | -0.104 | 1.818 | -0.057 | 0.954 |
| Routine: Component 5 | 0.342 | 1.390 | 0.246 | 0.806 |
| Going outside: Component 1 | -0.141 | 1.778 | -0.079 | 0.937 |
| Going outside: Component 2 | 0.114 | 0.938 | 0.122 | 0.903 |
| Going outside: Component 3 | 0.067 | 1.225 | 0.055 | 0.957 |
| Going outside: Component 4 | -0.427 | 1.131 | -0.378 | 0.705 |
| Balcony | -2.277 | 3.752 | -0.607 | 0.544 |
| Nicotine | 0.006 | 0.087 | 0.065 | 0.948 |
| Days in lockdown | 0.013 | 0.056 | 0.231 | 0.817 |
|  | | | | |
|  | **Estimate** | **Std. Err** | **z value** | **P (>\|z\|)** |
| DASS: Stress ~ | | | | |
| BF: Extraversion | -0.217 | 0.424 | -0.511 | 0.609 |
| BF: Agreeableness | 0.038 | 0.366 | 0.103 | 0.918 |
| BF: Conscientiousness | -0.242 | 0.328 | -0.736 | 0.462 |
| BF: Neuroticism | 0.733 | 0.499 | 1.468 | 0.142 |
| BF: Openness | 0.696 | 0.434 | 1.603 | 0.109 |
| FFMQ: Observation | -0.438 | 0.885 | -0.495 | 0.620 |
| FFMQ: Description | -1.271 | 1.369 | -0.929 | 0.353 |
| FFMQ: Aware actions | 1.111 | 1.051 | 1.057 | 0.290 |
| FFMQ: Non-judgment | 0.076 | 0.729 | 0.105 | 0.917 |
| FFMQ: Nonreactivity | -0.338 | 0.894 | -0.379 | 0.705 |
| Household | 1.529 | 1.232 | 1.241 | 0.215 |
| House size | -0.146 | 1.737 | -0.084 | 0.933 |
| Garden | -0.193 | 4.685 | -0.041 | 0.967 |
| Routine: Component 1 | 0.256 | 2.054 | 0.125 | 0.901 |
| Routine: Component 2 | -2.630 | 1.985 | -1.325 | 0.185 |
| Routine: Component 3 | 4.099 | 1.898 | 2.159 | 0.031 |
| Routine: Component 4 | 3.135 | 3.436 | 0.912 | 0.362 |
| Routine: Component 5 | -4.824 | 2.628 | -1.836 | 0.066 |
| Going outside: Component 1 | 2.204 | 3.360 | 0.656 | 0.512 |
| Going outside: Component 2 | 4.237 | 1.773 | 2.389 | 0.017 |
| Going outside: Component 3 | -1.740 | 2.315 | -0.751 | 0.452 |
| Going outside: Component 4 | -0.846 | 2.138 | -0.396 | 0.692 |
| Balcony | -9.107 | 7.090 | -1.284 | 0.199 |
| Nicotine | -0.107 | 0.165 | -0.647 | 0.517 |
| Days in lockdown | 0.170 | 0.105 | 1.616 | 0.106 |
|  | | | | |
|  | **Estimate** | **Std. Err** | **z value** | **P (>\|z\|)** |
| SCL-27: Total score~ | | | | |
| BF: Extraversion | -0.030 | 0.013 | -2.257 | 0.024 |
| BF: Agreeableness | 0.009 | 0.014 | 0.697 | 0.486 |
| BF: Conscientiousness | -0.026 | 0.012 | -2.223 | 0.026 |
| BF: Neuroticism | 0.006 | 0.017 | 0.335 | 0.737 |
| BF: Openness | 0.011 | 0.016 | 0.668 | 0.504 |
| FFMQ: Observation | 0.022 | 0.032 | 0.687 | 0.492 |
| FFMQ: Description | -0.001 | 0.050 | -0.018 | 0.986 |
| FFMQ: Aware actions | -0.106 | 0.043 | -2.496 | 0.013 |
| FFMQ: Non-judgment | 0.035 | 0.028 | 1.224 | 0.221 |
| FFMQ: Nonreactivity | -0.055 | 0.031 | -1.774 | 0.076 |
| **Household** | **0.187** | **0.050** | **3.768** | **< 0.001** |
| House size | -0.144 | 0.064 | -2.261 | 0.024 |
| Garden | -0.268 | 0.169 | -1.590 | 0.112 |
| Routine: Component 1 | -0.128 | 0.068 | -1.883 | 0.060 |
| **Routine: Component 2** | **-0.253** | **0.079** | **-3.225** | **0.001** |
| **Routine: Component 3** | **0.333** | **0.075** | **4.416** | **< 0.001** |
| Routine: Component 4 | -0.053 | 0.075 | -0.706 | 0.480 |
| Routine: Component 5 | -0.135 | 0.076 | -1.768 | 0.077 |
| Going outside: Component 1 | 0.042 | 0.081 | 0.513 | 0.608 |
| Going outside: Component 2 | 0.009 | 0.068 | 0.130 | 0.897 |
| Going outside: Component 3 | -0.189 | 0.081 | -2.344 | 0.019 |
| **Going outside: Component 4** | **-0.237** | **0.078** | **-3.042** | **0.002** |
| **Balcony** | **-0.562** | **0.212** | **-2.648** | **0.008** |
| Nicotine | -0.007 | 0.004 | -1.695 | 0.090 |
| **Days in lockdown** | **0.014** | **0.004** | **3.585** | **< 0.001** |
|  | | | | |
| **COVARIANCES** | | | | |
|  | **Estimate** | **Std. Err** | **z value** | **P (>\|z\|)** |
| DASS: Depression ~~ | | | | |
| **DASS: Anxiety** | **12.317** | **3.833** | **3.213** | **0.001** |
| **DASS: Stress** | **32.623** | **8.161** | **3.997** | **< 0.001** |
| DASS: Anxiety ~~ | | | | |
| **DASS: Stress** | **18.521** | **4.793** | **3.864** | **< 0.001** |
|  | | | | |
| **VARIANCES** | | | | |
|  | **Estimate** | **Std. Err** | **z value** | **P (>\|z\|)** |
| **DASS: Depression** | **32.906** | **7.651** | **4.301** | **< 0.001** |
| **DASS: Anxiety** | **11.913** | **2.770** | **4.301** | **< 0.001** |
| **DASS: Stress** | **42.547** | **9.892** | **4.301** | **< 0.001** |
| **SCL-27: Total score** | **0.061** | **0.014** | **4.359** | **< 0.001** |
|  | | | | |
| **R-SQUARE** | |  |  |  |
|  | **Estimate** |  |  |  |
| **DASS: Depression** | **0.659** |  |  |  |
| **DASS: Anxiety** | **0.366** |  |  |  |
| **DASS: Stress** | **0.541** |  |  |  |
| **SCL-27: Total score** | **0.745** |  |  |  |

## A-posteriori Power Analysis

In order to test the statistical power of our models (i.e., probability that the test correctly rejects the null hypothesis), we ran an a-posteriori power analysis. For this power analysis, we estimated the mean R^2^ from our models across all four cross-sectional surveys (mean R^2^ = 0.695) and the corresponding *f* ^2^ effect size, i.e., R^2^ / (1 - R^2^), which resulted in *f* ^2^ = 2.279. This value was used as the average effect size, the α significance level was set to 0.0125 as in our models, and the number of participants was set to 74, i.e., the lowest number of participants we collected from a cross-sectional survey, in order to obtain a conservative power estimate. The power analysis led to an estimated power > .999, thus supporting the statistical reliability of our results.
